# Global Estimates of the Prevalence and Incidence of Four Curable Sexually Transmitted Infections in 2012 Based on Systematic Review and Global Reporting

**DOI:** 10.1371/journal.pone.0143304

**Published:** 2015-12-08

**Authors:** Lori Newman, Jane Rowley, Stephen Vander Hoorn, Nalinka Saman Wijesooriya, Magnus Unemo, Nicola Low, Gretchen Stevens, Sami Gottlieb, James Kiarie, Marleen Temmerman

**Affiliations:** 1 Department of Reproductive Health and Research, World Health Organization, Geneva, Switzerland; 2 Consultant to Department of Reproductive Health and Research, World Health Organization, Geneva, Switzerland; 3 Statistical Consulting Centre, University of Melbourne, Melbourne, Australia; 4 WHO Collaborating Centre for Gonorrhoea and other STIs, Department of Laboratory Medicine, Örebro University Hospital and Örebro University, Örebro, Sweden; 5 Institute of Social and Preventive Medicine, University of Bern, Bern, Switzerland; 6 Department of Health Statistics and Information Systems, World Health Organization, Geneva, Switzerland; Fudan University, CHINA

## Abstract

**Background:**

Quantifying sexually transmitted infection (STI) prevalence and incidence is important for planning interventions and advocating for resources. The World Health Organization (WHO) periodically estimates global and regional prevalence and incidence of four curable STIs: chlamydia, gonorrhoea, trichomoniasis and syphilis.

**Methods and Findings:**

WHO’s 2012 estimates were based upon literature reviews of prevalence data from 2005 through 2012 among general populations for genitourinary infection with chlamydia, gonorrhoea, and trichomoniasis, and nationally reported data on syphilis seroprevalence among antenatal care attendees. Data were standardized for laboratory test type, geography, age, and high risk subpopulations, and combined using a Bayesian meta-analytic approach. Regional incidence estimates were generated from prevalence estimates by adjusting for average duration of infection. In 2012, among women aged 15–49 years, the estimated global prevalence of chlamydia was 4.2% (95% uncertainty interval (UI): 3.7–4.7%), gonorrhoea 0.8% (0.6–1.0%), trichomoniasis 5.0% (4.0–6.4%), and syphilis 0.5% (0.4–0.6%); among men, estimated chlamydia prevalence was 2.7% (2.0–3.6%), gonorrhoea 0.6% (0.4–0.9%), trichomoniasis 0.6% (0.4–0.8%), and syphilis 0.48% (0.3–0.7%). These figures correspond to an estimated 131 million new cases of chlamydia (100–166 million), 78 million of gonorrhoea (53–110 million), 143 million of trichomoniasis (98–202 million), and 6 million of syphilis (4–8 million). Prevalence and incidence estimates varied by region and sex.

**Conclusions:**

Estimates of the global prevalence and incidence of chlamydia, gonorrhoea, trichomoniasis, and syphilis in adult women and men remain high, with nearly one million new infections with curable STI each day. The estimates highlight the urgent need for the public health community to ensure that well-recognized effective interventions for STI prevention, screening, diagnosis, and treatment are made more widely available. Improved estimation methods are needed to allow use of more varied data and generation of estimates at the national level.

## Introduction

Sexually transmitted infections (STIs) are among the most common acute conditions in the world. There are over 30 infections that can be transmitted sexually. This paper focuses on four of the most common curable STIs–*Chlamydia trachomatis* (chlamydia), *Neisseria gonorrhoeae* (gonorrhoea), *Trichomonas vaginalis* (trichomoniasis), and *Treponema pallidum subspecies pallidum* (syphilis). These four infections cause acute conditions such as cervicitis, urethritis, and genital ulceration. They can also lead to severe complications and long term sequelae, including pelvic inflammatory disease, ectopic pregnancy, infertility, chronic pelvic pain and neurological and cardiovascular disease in adults, neonatal death, premature delivery, blindness, or severe disability in infants, and increased risk of HIV acquisition and transmission [1,2]. STIs also frequently result in stigma, stereotyping, vulnerability and shame, and have been associated with gender-based violence [3].

The World Health Organization (WHO) has produced estimates of the global and regional prevalence of four curable STIs (chlamydia, gonorrhoea, trichomoniasis and syphilis) approximately every 5 years since 1995 (Table 1) [4–7]. The majority of STI public health interventions are focused on these infections. Quantifying the prevalence and incidence of these STIs is important for planning and estimating the impact of program interventions and advocating for resources. In addition, these infections are curable so estimating incidence rates can provide insight into the potential impact of STI prevention and management strategies. The epidemiological and statistical methods used have changed over time so differences in the estimates between reports cannot be interpreted as trends over time.

**Table 1:** WHO estimates of new cases of chlamydia, gonorrhoea, trichomoniasis, and syphilis among adults for 1995, 1999, 2005, and 2008 using various methods [4 –7].

	Estimated number of new cases (millions)			
	1995	1999	2005	2008
**Chlamydia**	89	92	101	106
**Gonorrhoea**	62	62	88	106
**Trichomoniasis**	170	174	248	276
**Syphilis**	12	12	11	10

WHO bases its global and regional STI estimates on literature reviews of prevalence data among low risk or general populations and generates regional incidence estimates from the regional prevalence values. Three contextual changes have led to a need and an opportunity to change the WHO global estimation process for 2012. First, fewer STI prevalence studies amongst representative samples of the general population are being conducted and published, resulting in an increasing challenge to have sufficient number of data points to generate robust estimates. Second, in 2008 the HIV Universal Access/Global AIDS Response Progress Reporting (GARPR) system led by the WHO, Joint United Nations Programme on HIV and AIDS (UNAIDS), and United Nations Children's Fund (UNICEF), began to include the prevalence of syphilis among ANC attendees in the indicators routinely reported by countries [8]. Third, global standards for estimation as a whole have evolved, such that it is considered important to strive for country-level estimates (where data allow), and to incorporate measures of uncertainty [9].

This analysis was undertaken by WHO to generate global and regional prevalence and incidence estimates for 2012 for infection with the four curable STIs (chlamydia, gonorrhoea, trichomoniasis, and syphilis) for women and men aged 15–49 years age, based on prevalence data collected between 2005 and 2012.

## Methods

Methods to generate the 2012 estimates were based on those used to generate the 2005 and 2008 estimates [6,7]. There were three major changes that were implemented that arose from external expert consultations held by WHO in November 2013 and August 2014. First, the way in which countries were grouped to generate regional estimates for chlamydia, gonorrhoea and trichomoniasis was changed. In 2005 and 2008 countries were grouped into WHO regions with two regions subdivided (Africa and the Western Pacific) [6]. For the 2012 estimates, we defined 10 regions based on those used by the Global Burden of Disease project 2010 [10] and on epidemiology, geography and data availability (S1 Map). Second, syphilis estimates were based on country level data from the GARPR system; third, we used a Bayesian meta-analytic approach to generate the estimates and uncertainty around them.

### Estimating prevalence: chlamydia, gonorrhoea, and trichomoniasis

**Fig 1:**
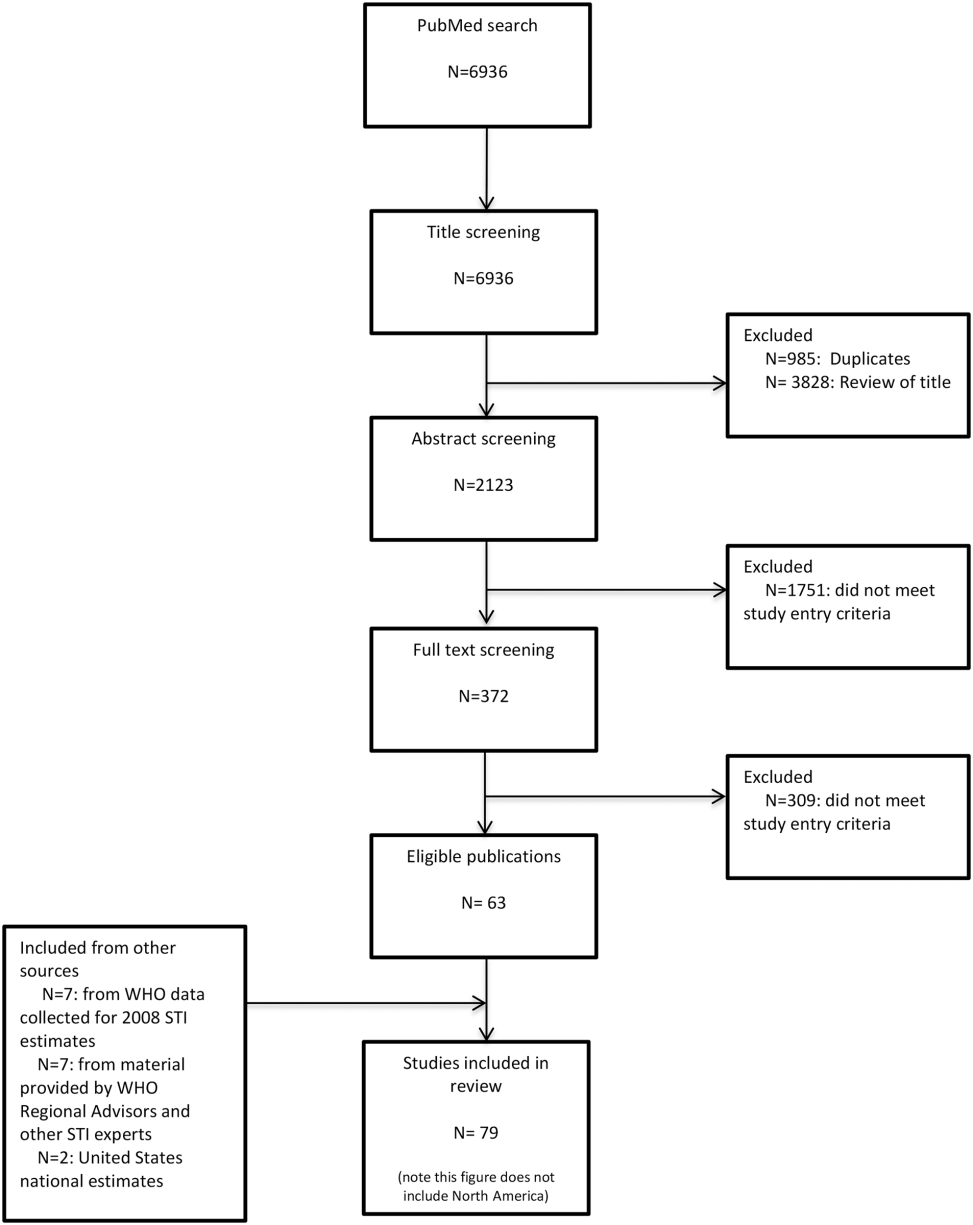
PRISMA flowchart.

Prevalence data for chlamydia, gonorrhoea, and trichomoniasis were drawn from the data collected for the 2008 WHO estimates, PubMed literature searches (last search conducted on January 30, 2015) (S1 Text) and requests to the WHO regional STI advisors and other leading experts in the field. In addition, members of the International Union against STIs were asked to identify additional published or unpublished studies that met the study inclusion criteria. Two investigators applied inclusion criteria: sample size of at least 100; specimens collected from 2005 through 2012 (for studies in which no specimen collection date was specified the study had to be published in 2006 or later); population could be considered representative of the general population (study populations included pregnant women, women at delivery, women attending family planning clinics, military recruits, or individuals selected for participation in a Demographic and Health Survey); and study used an internationally recognised diagnostic test with adequate performance characteristics on urine, urethral, or cervicovaginal specimens. Studies conducted among the following groups were excluded because of reasons that are known to bias estimates of general population STI prevalence: patients seeking care for an STI or genital symptoms, women with abnormal Papanicolaou smears, blood donors, women attending gynaecology or sexual health clinics, remote or indigenous populations, men who have sex with men, and commercial sex workers. Duplicate data points were removed and if data were published in more than one paper the paper with most information was included in the database (Fig 1). PRISMA guidance was followed for the manuscript (S2 Text).

Data from studies that met the inclusion criteria (S1 Data) were standardised to approximate the prevalence in the general population of a country by applying adjustment factors for the laboratory diagnostic test used, study location (rural vs. urban) and the age of the study population (S3 Text). In order to reflect the contribution of populations at higher risk of infection, the regional prevalence estimates were increased by 10% for males and females for all four infections. The 10% figure was based on the estimate used in the 2005/08 WHO estimates [6,7] and is a rough approximation of the contribution of these populations.

For some infections, particularly in men, there were fewer than three data points per region for the 2005 to 2012 time period. When there were insufficient data for men, the prevalence in men was assumed to be a proportion of the estimated prevalence in women. This proportion was assumed to be infection-specific, but to be the same across all regions and derived from a uniform distribution with a range of +/- 33.3% of the values used in the 2005 and 2008 estimates (Table 2).

**Table 2:** Ratios used to estimate prevalence when data for chlamydia, gonorrhoea, or trichomoniasis were limited.

Ratio	Infection/region	Estimated ratio	Range	Comments
**Male to female STI ratios—used when fewer than three data points for a men with a particular infection (same values used for all regions)**	Chlamydia	0.8	0.53–1.07	Used the ratio estimated for the 2005 and 2008 [6,7]
	Gonorrhoea	0.86	0.58–1.15	Used the ratio estimated for the 2005 and 2008 [6,7]
	Trichomoniasis	0.1	0.07–0.13	Used the ratio estimated for the 2005 and 2008 [6,7]
**Gonorrhoea to chlamydia ratio in females—used when fewer than three gonorrhoea data points**	Western, Central & Eastern Europe, Central Asia	0.16	0.11–0.21	Estimate based on gonorrhoea to chlamydia ratio from published estimates for High Income North America [11,12]
	East & South East Asia	0.21	0.14–0.28	Estimate based on the median of the ratio of gonorrhoea to chlamydia ratio from all studies with data on both gonorrhoea and chlamydia. Studies where prevalence was zero for either infection was 0 were excluded (S1 Data)
**Trichomoniasis to chlamydia ratio in females—used when fewer than three trichomoniasis data points**	Western, Central & Eastern Europe, Central Asia, Australasia, High Income Asia Pacific	0.2	0.13–0.27	Estimate based on a review of studies in Europe presenting prevalence data for trichomoniasis in various populations, including STI patients [13–17]
	Oceania, South Asia	1.20	0.80–1.60	Estimate based on the median of the ratio of trichomoniasis to chlamydia ratio from all studies with data on both trichomoniais and chlamydia. Studies where prevalence was zero for either infection was 0 were excluded (S1 Data)

For women there were sufficient data points to generate estimates for all regions for chlamydia but not for gonorrhoea or trichomoniasis. In the absence of data, a ratio was assumed between the prevalence of these two infections and chlamydia. The estimated ratio was derived from a uniform distribution within a range of +/- 33.3% (Table 2).

The estimates for the High Income North America region (United States of America and Canada) were based on the United States national chlamydia estimates for 2012 and the gonorrhoea and trichomoniasis estimates for 2008 published by the United States Centers for Disease Control and Prevention (CDC) [11,12]. We assumed that the prevalence of gonorrhoea and of trichomoniasis did not change from 2008 to 2012 in the United States, that the prevalence of each infection in Canada was the same as in the United States, and that their data (calculated for a 15–39 year age range) could be extrapolated to the 15–49 year age range used for the WHO estimates. The geography, age, and high risk adjustments used for other regions in the 2012 WHO estimates process were not applied (S3 Text).

### Estimating prevalence: syphilis

Syphilis prevalence estimates were based on the WHO 2012 estimates of maternal and congenital syphilis [18] reported through the GARPR system for 122 of 194 (63%) countries accounting for 76% of individuals aged 15–49 years globally. A PubMed search of the peer reviewed literature conducted in March 2014 identified data for an additional 8 (4.1%) countries missing GARPR data. Where estimates were available from both peer-reviewed literature and GARPR, we used the GARPR estimate because Ministry of Health officials provide the most nationally representative data available. Such national surveillance data are typically not published in the peer-review literature.

Syphilis prevalence estimates were calculated at country level before aggregating for regional and global estimates. All reported data were adjusted for laboratory test to approximate the prevalence of “probable active” infections, defined as having positive serology on both treponemal and non-treponemal tests [19]. We assumed that the prevalence of syphilis in women aged 15–49 years was the same in pregnant and non-pregnant women. In addition, we assumed that the ratio of the prevalence of syphilis in males to females was 1 with a range of +/- 33.3%. As in the 2005 and 2008 estimates, the regional syphilis prevalence estimates for men and women were increased by 10% to reflect the contribution of key high risk populations.

### Statistical methods used to pool data

We used a Bayesian meta-analytic approach produce pooled prevalence estimates for the 10 regional groups where there were three or more data points for a particular infection in women or men. The beta-binomial approach for pooling over-dispersed binomial data was used as it provides a robust estimate for the average proportion based on data from heterogeneous studies [20]. The beta-binomial model is appropriate for meta-analysis of prevalence data when the outcome is rare, as for gonorrhoea and syphilis in some regions, and provides estimates of uncertainty that take into account the heterogeneity observed between studies [21].

Meta-analyses were performed with a Markov Chain Monte Carlo (MCMC) algorithm implemented with the BRrugs package [22]. For chlamydia, gonorrhoea, and trichomoniasis, ten thousand samples were generated from the posterior distribution for the expected mean prevalence of each regional grouping separately based on the beta-binomial model and these were used to calculate uncertainty intervals (UI) given by the 2.5 and 97.5 percentiles. Uncertainty, calculated under this approach, reflects both the level of precision within studies (i.e. sample size) and also additional between-study variation arising either from real differences between study populations or differences in study methodology. Global prevalence estimates for chlamydia, gonorrhoea and trichomoniasis were then calculated by weighting the regional prevalence estimates according to population size. The same approach was applied for syphilis, but country level data were used and pooled within each of the Global Burden of Disease project regions [1]. For syphilis, uncertainty was quantified based on analysis of the posterior expected value for country level prevalence and global prevalence calculated by weighting according to country population size. All analyses were carried out using R statistical software [23].

### Estimating incidence: chlamydia, gonorrhoea, trichomoniasis, and syphilis

Regional incidence estimates for nine of the 10 regions were estimated from prevalence estimates. The approach was the same as in 2005 and 2008 but we also applied a range of +/- 33.3% around the estimated duration of infection to reflect uncertainty when calculating incidence using the equation: incidence = prevalence/average duration of infection. For the High Income North America region we used incidence estimates for chlamydia, gonorrhoea and trichomoniasis based on the United States national incidence estimates [11,12].

It was assumed that the average duration of infection in each region varies according to the average duration of infection in the absence of treatment for symptomatic and asymptomatic individuals and the probability symptomatic and asymptomatic individuals are treated appropriately. For the 2005 and 2008 estimates countries were classified into one of three treatment groups with a corresponding average duration of infection based on a literature review and expert consultation [6]. A review of the literature in 2012 found insufficient data to merit changing the assumptions for duration of infection used in 2005 and 2008 (S4 Text).

Prevalence and incidence estimates from the 10 regions were aggregated so that results could be presented by WHO regional groupings and by World Bank income classification [24]. United Nations Population data for women and men aged 15–49 years were used to generate numbers of prevalent and incident cases [25].

## Results

The search strategy identified 63 studies with data that met the study inclusion criteria for one or more of the three infections. An additional 16 studies were identified through other means for a total of 79 unique studies used for analysis (S1 Data and S1 References). Extracted from these studies were the following: 70 data points for chlamydia in women and 22 in men, 51 for gonorrhoea in women and 13 in men, and 45 for trichomoniasis in women and two in men (Table 3). Forest plots for the unadjusted reported data from included studies by infection, grouped according to WHO region were produced (S1 Figs A3.1-A3.6). The data were heterogeneous, particularly for chlamydia and gonorrhoea in women in some regions.

**Table 3:** Number of data points that met the study inclusion criteria for WHO 2012 estimates by infection, region, and sex. Note: A total of 79 studies were identified, but only some provided information for all three infections. For the High Income North America region, the estimates were based on the United States national estimates.

Regional grouping	Chlamydia		Gonorrhoea		Trichomoniasis	
	Women	Men	Women	Men	Women	Men
**Central, Eastern & Western Sub-Saharan Africa**	14	3	13	3	13	0
**Southern Sub-Saharan Africa**	11	0	11	0	10	0
**Andean, Central, Southern & Tropical Latin America and Caribbean**	10	3	5	1	5	0
**High Income North America**	1	1	1	1	1	1
**North Africa & Middle East**	6	1	4	0	5	0
**Australasia & High Income Asia Pacific**	8	4	3	2	2	1
**Western, Central & Eastern Europe and Central Asia**	5	5	2	1	1	0
**Oceania**	8	0	8	0	1	0
**South Asia**	4	4	3	4	0	0
**East Asia and South East Asia**	3	1	1	1	7	0
**Global number of studies**	**70**	**22**	**51**	**13**	**45**	**2**

**Fig 2:**
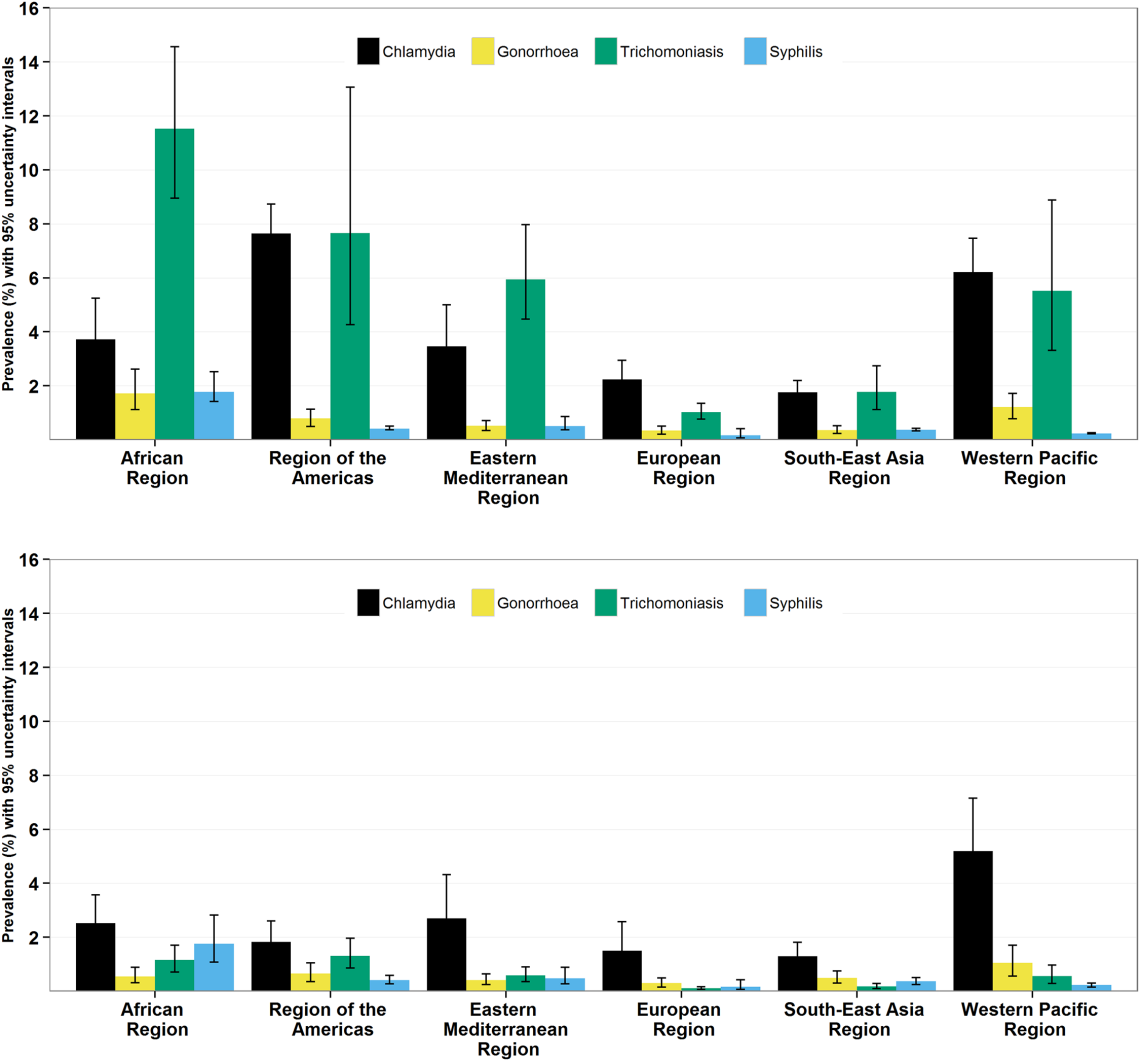
Estimated prevalence (and 95% UI) of chlamydia, gonorrhoea, trichomoniasis, and syphilis in women and men aged 15–49 years by WHO region, based on 2005–2012 data.

Based on data for 2005 to 2012, the estimated pooled prevalence for chlamydia in women globally was 4.2% (95% UI: 3.7–4.7%), with regional values ranging from 1.8% to 7.6% (S1 Figs). For gonorrhoea the global estimate was 0.8% (0.6–1.0%) and the regional values ranged from 0.3% to 1.7%, for trichomoniasis the global estimate was 5.0% (4.0–6.4%) and the regional values ranged from 1.0% to 11.5%, and for syphilis the global estimate was 0.5% (0.4–0.6%) and the regional values ranged from 0.2% to 1.8% (Fig 2 and Table A in S1 Tables). Prevalence estimates for chlamydia were highest in the Region of the Americas and Western Pacific Region. For the other STIs, the highest estimated prevalence rates were in the African Region. Uncertaintywas greatest for estimates of trichomoniasis prevalence.

The estimated pooled prevalence for chlamydia in men globally was 2.7% (95% UI: 2.0–3.6%), with regional values that ranged from 1.3% to 5.2% (Fig 2 and S1 Tables). For gonorrhoea the global estimate was 0.6% (0.4–0.9%) and the regional values ranged from 0.3% to 1.0%, for trichomoniasis the global estimate was 0.6% (0.4–0.8%) and the regional values ranged from 0.1% to 1.3%, and for syphilis the global estimate was 0.5% (0.3–0.7%) and the regional values ranged from 0.2% to 1.8% (Fig 2 and Table A in S1 Tables). For chlamydia and gonorrhoea, the highest estimated prevalence rates were in the Western Pacific Region. Estimated syphilis prevalence was highest in the African Region and estimated trichomoniasis prevalence was highest in the Region of the Americas and African Region. Uncertainty was greatest for estimates of chlamydia prevalence.

Applying these prevalence rates to 2012 population, there were an estimated 127,361,000 prevalent cases of chlamydia, 26,819,000 prevalent cases of gonorrhoea, 100,988,000 prevalent cases of trichomoniasis, and 17,721,000 prevalent cases of syphilis in men and women aged 15–49 years (Table 4).

**Table 4:** Global and regional estimates of the number of prevalent cases (thousands) in 2012 by infection and sex (95% UI shown in parentheses).

	Region	Chlamydia	Gonorrhoea	Trichomoniasis	Syphilis
**Females**	African Region	7,833 (5,622–11,061)	3,616 (2,355–5,510)	24,284 (18,870–30,706)	3,723 (2,981–5,302)
	Region of the Americas	18,805 (16,400–21,473)	1,936 (1,205–2,785)	18,836 (10,495–32,132)	1,003 (888–1,239)
	Eastern Mediterranean Region	5,466 (3,821–7,892)	809 (533–1,116)	9,361 (7,049–12,562)	792 (590–1,353)
	European Region	4,928 (3,628–6,514)	744 (464–1109)	2,254 (1,671–2,983)	366 (157–898)
	South-East Asia Region	8,498 (6,738–10,612)	1,733 (1,109–2,492)	8,519 (5,394–13,185)	1,791 (1,580–2,021)
	Western Pacific Region	30,524 (24,934–36,713)	5,949 (3,834–8,450)	27,128 (16,274–43,699)	1,109 (1,046–1,249)
	**Global total (females)**	**76,054 (67,654–85,608)**	**14,787 (11,432–18,700)**	**90,381 (71,761–115,083)**	**8,783 (7,761–10,645)**
**Males**	African Region	5,328 (3,499–7,544)	1,163 (656–1,880)	2,440 (1,510–3,590)	3,726 (2,268–5,959)
	Region of the Americas	4,451 (3,067–6,322)	1,584 (864–2,538)	3,193 (2,081–4,777)	992 (650–1,419)
	Eastern Mediterranean Region	4,593 (2,706–7,352)	706 (411–1,089)	998 (607–1,522)	816 (465–1,498)
	European Region	3,334 (1,931–5,732)	656 (345–1095)	238 (144–362)	368 (141–937)
	South-East Asia Region	6,588 (4,344–9,203)	2,475 (1,505–3,820)	864 (457–1,458)	1,856 (1,241–2,544)
	Western Pacific Region	27,012 (18,286–37,269)	5,448 (2,898–8,898)	2,874 (1,472–5,064)	1,179 (795–1,584)
	**Global total (males)**	**51,307 (37,693–66,666)**	**12,033 (7,812–17,345)**	**10,606 (7,137–14,982)**	**8,938 (5,865–12,638)**

The estimated prevalence of the four curable STI varied by infection and World Bank classification (Table 5). The prevalence of chlamydia among women ranged from 2.4% to 6.9%, gonorrhoea from 0.3% to 1.2%, trichomoniasis from 1.9% to 7.8%, and syphilis from 0.2% to 1.3%. The prevalence of chlamydia among men ranged from 1.6% to 4.2%, gonorrhoea from 0.3% to 1.0%, trichomoniasis from 0.3% to 0.8%, and syphilis from 0.2% to 1.3%. A consistent pattern was seen for syphilis in women, with increasing prevalence by decreasing income.

**Table 5:** Prevalence by infection and World Bank classification. Note: Estimates by World Bank classification are not identical to those by geographic region as three countries are not classified by World Bank and were therefore excluded: Cook Islands, Niue, and Nauru.

	Classification	Chlamydia	Gonorrhoea	Trichomoniasis	Syphilis
**Females**	High-income economies	3.0%	0.3%	1.9%	0.2%
	Upper-middle income economies	6.9%	1.2%	6.9%	0.3%
	Lower-middle income economies	2.4%	0.6%	3.5%	0.5%
	Low-income economies	2.9%	1.1%	7.8%	1.3%
	**Female total**	**4.2%**	**0.8%**	**5.0%**	**0.5%**
**Males**	High-income economies	2.4%	0.3%	0.6%	0.2%
	Upper-middle income economies	4.2%	1.0%	0.7%	0.3%
	Lower-middle income economies	1.6%	0.5%	0.3%	0.5%
	Low-income economies	2.0%	0.5%	0.8%	1.3%
	**Male total**	**2.7%**	**0.6%**	**0.6%**	**0.5%**

**Fig 3:**
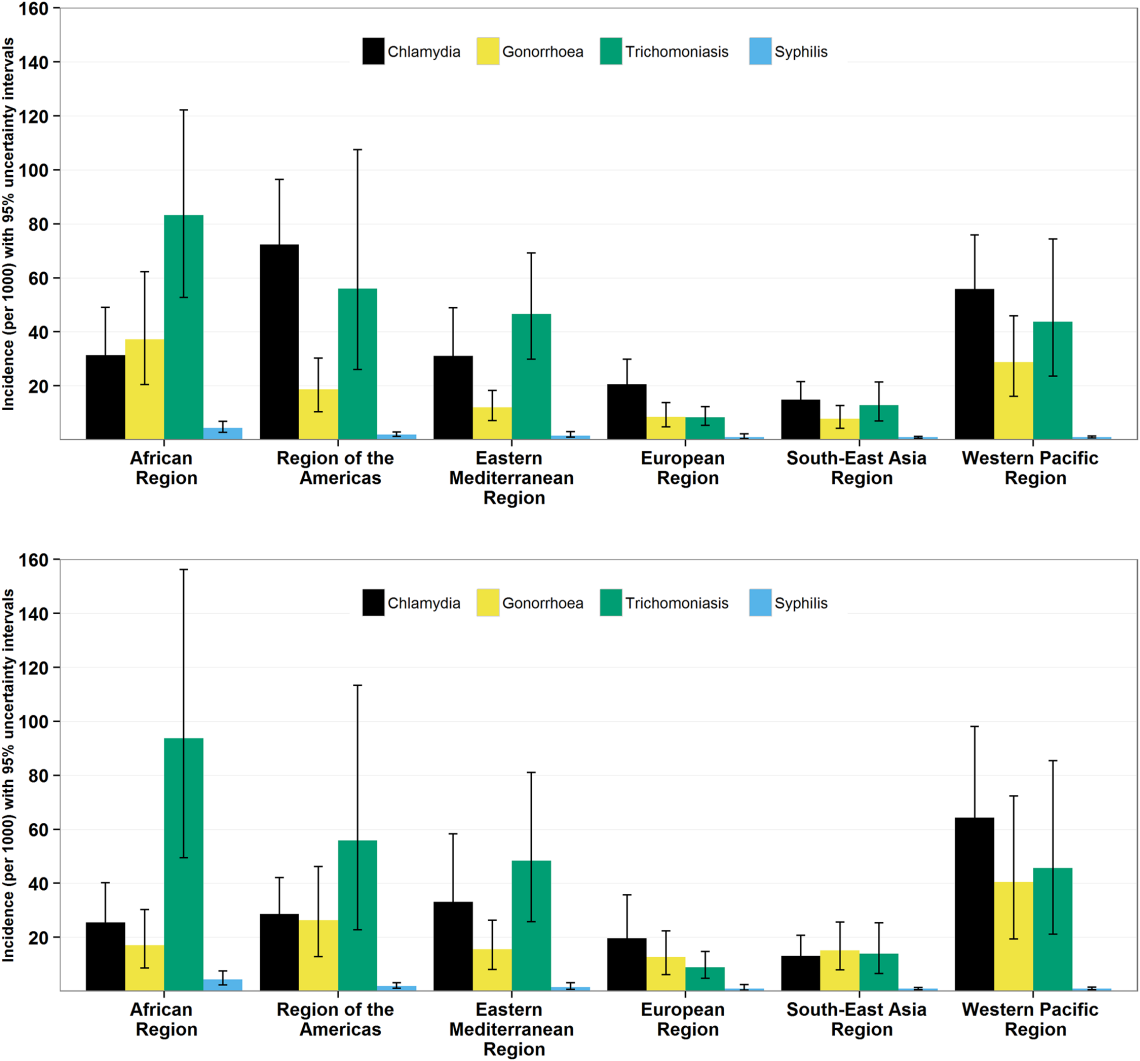
Incidence (and 95% UI) of chlamydia, gonorrhoea, trichomoniasis, and syphilis in women and men aged 15–49 years by WHO region, based on 2005 to 2012 data.

The global incidence rate for chlamydia was estimated to be 38 per 1,000 in women (regional range: 15–72) and 33 per 1,000 in men (regional range: 13–64); for gonorrhoea the rate was 19 cases per 1,000 in women (regional range: 8–37), and 24 per 1,000 in men (regional range: 13–41), for trichomoniasis the rate was 38 cases per 1,000 in women (regional range: 8–83), and 40 per 1,000 in men (regional range: 9–94), and for syphilis 1.5 cases per 1,000 in women (regional range: 0.9–4.4) and 1.5 per 1,000 in men (regional range: 0.9–4.4) (Fig 3 and Table B in S1 Tables). Amongst women, regional patterns of incidence were the same as those for prevalence. Amongst men, trichomoniasis was estimated to be the most common incident infection.

These incidence rates translated into 131 million new cases of chlamydia, 78 million of gonorrhoea, 143 million of trichomoniasis, and 5.6 million of syphilis in women and men aged 15–49 years in 2012 (Table 6).

**Table 6:** Global and regional estimates of the number of incident cases (in thousands) in 2012 by infection and sex (95% uncertainty interval shown in parentheses).

Sex	Region	Chlamydia	Gonorrhoea	Trichomoniasis	Syphilis
Females	African Region	6,616 (4,020–10,337)	7,833 (4,312–13,117)	17,529 (11,115–25,763)	920 (578–1,433)
	Region of the Americas	17,775 (12,348–23,729)	4,594 (2,531–7,440)	13,776 (6,398–26,426)	471 (309–696)
	Eastern Mediterranean Region	4,893 (2,923–7,716)	1,885 (1,112–2,871)	7,348 (4,702–10,909)	243 (146–481)
	European Region	4,554 (2,940–6,581)	1,877 (1,060–3,048)	1,842 (1,170–2,708)	219 (92–495)
	South-East Asia Region	7,168 (4,634–10,373)	3,769 (2,046–6,128)	6,151 (3,335–10,298)	435 (290–596)
	Western Pacific Region	27,449 (18,931–37,330)	14,138 (7,922–22,546)	21,474 (11,577–36,585)	482 (332–650)
	**Global total (females)**	**68,455 (50,649–87,648)**	**34,095 (23,309–47,622)**	**68,120 (46,632–95,620)**	**2,769 (2,006–3,707)**
Males	African Region	5,400 (3,098–8,510)	3,607 (1,817–6,398)	19,831 (10,462–33,096)	923 (481–1,597)
	Region of the Americas	6,960 (4,487–10,221)	6,380 (3,114–11,214)	13,593 (5,533–27,560)	466 (253–770)
	Eastern Mediterranean Region	5,634 (2,905–9,937)	2,641 (1,365–4,485)	8,241 (4,389–13,814)	253 (123–528)
	European Region	4,375 (2,306–7,961)	2,809 (1,364–4,988)	1,974 (1,053–3,284)	221 (85–534)
	South-East Asia Region	6,621 (3,682–10,471)	7,638 (3,982–12,962)	7,021 (3,307–12,879)	451 (243–720)
	Western Pacific Region	33,487 (20,202–51,084)	21,109 (10,104–37,673)	23,778 (10,978–44,515)	511 (286–804)
	**Global total (males)**	**62,477 (41,456–88,926)**	**44,185 (25,880–69,519)**	**74,438 (42,001–118,832)**	**2,825 (1,665–4,342)**

Approximately 56% of these infections occurred in upper-middle income, 23% in lower-middle income, 12% in low-income and 9% in high-income countries (Table 7).

**Table 7:** Number of incident cases (‘000) in 2012 by infection and World Bank income classification. Note: Estimates by World Bank classification are not identical to those by geographic region as three countries are not classified by World Bank and were therefore excluded: Cook Islands, Niue, and Nauru.

Sex	Classification	Chlamydia	Gonorrhoea	Trichomoniasis	Syphilis
Females	High-income economies	9,109	2,525	3,194	374
	Upper-middle income economies	5,168	5,214	11,843	695
	Lower-middle income economies	13,465	7,858	16,753	879
	Low-income economies	40,712	18,498	36,329	822
	**Female total**	**68,455**	**34,095**	**68,120**	**2,769**
Males	High-income economies	11,423	3,707	2,999	389
	Upper-middle income economies	4,203	2,986	13,330	689
	Lower-middle income economies	11,701	10,432	18,869	905
	Low-income economies	35,149	27,060	39,240	842
	**Male total**	**62,477**	**44,185**	**74,438**	**2,825**

## Discussion

### Summary of findings

This study suggests that, in 2012, there were about 273 million prevalent cases of the four curable STI among adults aged 15–49 years: 128 million cases of chlamydia, 27 million cases of gonorrhoea, 101 million cases of trichomoniasis, and 18 million cases of syphilis. These figures correspond to an estimated incidence of 131 million new chlamydia cases, 78 million new gonorrhoea cases, 143 million new trichomoniasis cases, and 5.6 million new syphilis cases in women and men aged 15–49 years globally. Together the four infections accounted for 357 million new infections in adults that year, or an average of nearly one million new infections each day. Because STI co-infection is common, however, it is unclear exactly how many individuals this represents.

### Strengths and limitations

The methods used to generate the 2012 estimates of STI prevalence were based on the methods used for the 2005 and 2008 estimates, with modifications to improve the quality of the estimates based on input from external expert consultations. In particular, the prevalence data were pooled into 10 regions based on epidemiological similarity, geography and data availability rather than by geography alone and greatly improved data for syphilis in women were available at country level through the GARPR system.

The biggest challenges to estimates of this nature are the quantity, representativeness and risk of bias of the available data. Data were included for an eight year time window (2005 through 2012), which means that changes in prevalence over time within this window will adversely affect the accuracy of estimates for 2012, a single year. However, even with an 8 year time window the number of data points for each infection was limited, particularly for men; only two usable data points were identified for trichomoniasis in men. As a result, the authors applied adjustment factors to prevalence estimates derived from available data for all four STIs in men and for gonorrhoea and trichomoniasis in women. The current estimates are limited to urogenital infections. Gonorrhoea and chlamydia can also be rectal and oropharyngeal infections; however, data on their prevalence is even more limited.

Where data were available, estimates of STI prevalence varied substantially. Between-study heterogeneity could represent true variation in infection prevalence between different populations but could also result from methodological sources of bias. For example, the response rate in chlamydia prevalence surveys in high income countries is inversely associated with the estimated prevalence [26]. Pooling data is not ideal under such circumstances but was necessary to generate estimates at regional and global levels. The Bayesian approach, using a beta-binomial model was deemed the most appropriate method for meta-analysis, given the challenges arising from the data collection [20,21]. The over-dispersion parameter in the model made an allowance for the between-study heterogeneity, which is reflected in the uncertainty intervals around the mean prevalence. The estimated incidence rates were derived indirectly, based on the relationship between prevalence and the duration of infection, owing to the absence of good quality incidence data from empirical studies. Assumptions were made about the duration of infection for women and men, levels of symptomatic and asymptomatic infection, all of which vary widely within and between countries. The 2012 duration estimates are unchanged from the 2005 and 2008 estimates although we included a range of values to reflect some of the uncertainty around these estimates.

### Comparison with other studies

The Global Burden of Disease 2013 study included morbidity estimates of chlamydia, gonorrhoea, and trichomoniasis, and for tertiary but not primary or secondary syphilis [1]. The Global Burden of Disease study estimated that there were 130 million new chlamydia cases, 74 million new gonorrhoea cases, and 55 million trichomoniasis cases among adults aged 15 to 49 years in 2013. The figures for chlamydia and gonorrhoea were very similar to the WHO estimates and well within the WHO uncertainty intervals. The difference in the trichomoniasis estimates is primarily driven by the high number of new cases in men estimated by WHO (143 million). The high incidence of trichomoniasis in men in the WHO estimates results from the duration of asymptomatic infection, which was assumed to be 1.5 months in men and 18 months in women. The Global Burden of Disease process did not vary presence of symptoms or duration of infection by gender, nor did it include standardization for laboratory test performance. However, for any global estimation process, improved data on trichomoniasis among men will be critical for improving the robustness of the estimates. The estimated prevalence of chlamydia in high income countries is in line with estimates from a published systematic review [26].

### Interpretation of the findings

The 2012 global estimates of STI prevalence and incidence are not directly comparable with earlier estimates and are not appropriate for doing trend analyses, owing to differences in the methods used for each set of estimates. In 2012 the estimate for the number of new cases for the four infections combined was 357 million. The corresponding figures for 2005 and 2008 were 448 and 498 million, respectively. The empirical data for syphilis are the most robust and do suggest decreases over time. A recent analysis of the ANC syphilis seropositivity data on which the current estimates were based was able to document a decrease in syphilis seropositivity among consistently reporting countries [18]. For the other infections it is not possible to say how much of the overall decrease reflects changes in the representativeness of the data, changes in the methods used for analysis, or true decreases in infection.

STIs are a global epidemic but there are distinct regional variations. The highest prevalence and incidence rates for syphilis were found in the WHO African Region. Access to antenatal syphilis screening contributes to regional differences in syphilis prevalence. In the African region, the median reported proportion of ANC attendees tested for syphilis was 58%. The median proportions were markedly higher in the other regions and ranged from 83 to 99% [27]. Of note, estimated chlamydia prevalence was relatively low in the African Region and highest in the Region of the Americas and the Western Pacific in women and in the Western Pacific in men. The substantial unexplained heterogeneity between studies of chlamydia prevalence across and within regions deserves further detailed investigation. Both prevalence and incidence of all four infections were lowest in the European and South-East Asian regions. Possible contributing factors are a lower risk of infection due to lower numbers of partners and increased condom use, or better social and economic conditions including clinical STI services. Alternatively, data from the 2005 to 2012 time period might not be representative of these regions.

In all regions, the estimated prevalences of chlamydia in women and men, and of trichomoniasis in women were greater than gonorrhoea or syphilis prevalence. There are several potential factors contributing to these findings. Repeat infection with both trichomoniasis and chlamydia are quite common following currently recommended single dose STI treatment regimens, often because partners are not adequately treated [28]. Additionally, most infections with chlamydia and trichomoniasis in women are asymptomatic and remain untreated [2]. Because most men with gonorrhoea are symptomatic, syndromic management has been effective in decreasing gonorrhoea burden. The higher frequency of asymptomatic STIs in women compared with men, resulting in a longer average duration of infection contributes to the finding that over 69% of the prevalent STI cases but only 48% of the new STI cases were among women.

When looking at incidence by World Bank income classification, 91% of incident infections were among individuals in low, lower-middle, and upper-middle income countries where 84% of the population aged 15 to 49 years live. Over half (56%) of all incident infections were in upper-middle income countries where only 36% of the population aged 15–49 years lives. The prevalence of chlamydia and of gonorrhoea in upper-middle income countries were strikingly high, but it is unclear whether this was an artefact of variable data or whether this was a true epidemiological finding due to differences in sexual risk behaviour or access to care. For syphilis in women, the infection with the most robust available data, the prevalence of infection decreased as average country income increased. This trend also held for gonorrhoea and trichomoniasis, apart from the data points for upper-middle income countries.

### Implications of the findings

WHO and its partners are looking at options to improve the quality of future estimates and supporting countries to generate their own national estimates. More data from studies conducted amongst women and men at low risk of infection in the general population are needed to improve estimates of STI prevalence. For example, South Asia, South East and East Asia Regions together had only 10 prevalence data points for chlamydia, gonorrhoea, and trichomoniasis that met the inclusion criteria, yet these three regions account for over half (54%) of the world population aged 15–49 years. Similarly, there were only nine data points for Western, Central, and Eastern Europe and Central Asia, all of which were for chlamydia. For many countries, national STI reference laboratories and surveillance systems need to be strengthened. WHO is also exploring alternative methods for generating STI prevalence and incidence estimates including approaches that use data collected from studies done in key populations, case reports, and data from routine programmes. Robust estimates of the size of key populations such as commercial sex workers and men who have sex with men will also be needed. Statistical methods such as hierarchical Bayesian modelling [20] can provide the tools to combine and explore sources of uncertainty in data from different sources.

In conclusion, the global prevalence and incidence of urogenital chlamydia, gonorrhoea, trichomoniasis, and syphilis in adult men and women aged 15 to 49 years remain high, with nearly one million new cases of curable STI acquired each day. The global threat of antimicrobial resistance, particularly for gonorrhoea [18], makes it essential to improve the monitoring of changes in STI incidence. Robust estimates of STI prevalence and incidence data are important for designing, implementing and evaluating STI interventions and for advocating for funding for program and research efforts to develop improved tools such as more efficacious therapeutics, point-of-care diagnostics, vaccines and microbicides appropriate for global use.

## Supporting Information

S1 Data Table of the chlamydia, gonorrhoea and trichomoniasis data from the current literature review and included from previous reviews that met the study entry criteria. (XLSX)

S1 Map Classification of countries for WHO 2012 estimates of chlamydia, gonorrhoea, trichomoniasis, and syphilis. (AI)

S1 Figs Forest plots for unadjusted reported data on chlamydia, gonorrhoea, and trichomoniasis in women and men, grouped by World Health Organization region. (TIFF)

S1 References List of publications to accompany S1 Data and S1 Figs. (DOCX)

S1 Tables Global and regional estimates for 2012 by infection and sex of the percentage of population with prevalent infection and the estimated incidence rates (per 1,000). (DOCX)

S1 Text Search strategy. (DOCX)

S2 Text PRISMA 2009 checklist. (DOC)

S3 Text Standardizing prevalence data across studies. (DOCX)

S4 Text Estimating duration of infection. (DOCX)

S5 Text WHO copyright permission S1 Map. (PDF)
