# Supplementary material for: Global Estimates of the Prevalence and Incidence of Four Curable Sexually Transmitted Infections in 2012 Based on Systematic Review and Global Reporting
Source: PLoS One. 2015 Dec 8;10(12):e0143304. doi: 10.1371/journal.pone.0143304 (PMC4672879; doi:10.1371/journal.pone.0143304)
Supplement: S1 References — (DOCX) [file pone.0143304.s004.docx]

**S1 References**

| 1 | Béhanzin L, Diabaté S, Minani I, Lowndes CM, Boily M-C, Labbé A-C, et al. Decline in HIV prevalence among young men in the general population of Cotonou, Benin, 1998-2008. PLoS One [Internet]. Public Library of Science; 2012 Jan 28 [cited 2015 Jun 22];7(8):e43818. Available from: http://journals.plos.org/plosone/article?id=10.1371/journal.pone.0043818 |
| --- | --- |
| 2 | Eshete A, Mekonnen Z, Zeynudin A. Trichomonas vaginalis Infection among Pregnant Women in Jimma University Specialized Hospital, Southwest Ethiopia. ISRN Infect Dis [Internet]. 2013;2013(Article ID 485439):1–5. Available from: http://www.hindawi.com/journals/isrn.infectious.diseases/2013/485439/ |
| 3 | Yirenya-Tawiah D, Annang TN, Apea-Kubi KA, Lomo G, Mensah D, Akyeh L, et al. Chlamydia Trachomatis and Neisseria Gonorrhoeae prevalence among women of reproductive age living in urogenital schistosomiasis endemic area in Ghana. BMC Res Notes [Internet]. 2014 Jan [cited 2015 Jun 11];7(1):349. Available from: http://www.biomedcentral.com/1756-0500/7/349 |
| 4 | Drake AL, Kinuthia J, Matemo D, McClelland RS, Unger J, John-Stewart G. P3.079 Prevalence and Cofactors For STIs Among Pregnant Adolescents in Western Kenya. Sex Transm Infect [Internet]. 2013 Jul 13 [cited 2015 Jun 22];89(Suppl 1):A172–A172. Available from: http://sti.bmj.com/content/89/Suppl_1/A172.3.abstract |
| 5 | Otieno FO, Ndivo R, Oswago S, Pals S, Chen R, Thomas T, et al. Correlates of prevalent sexually transmitted infections among participants screened for an HIV incidence cohort study in Kisumu, Kenya. Int J STD AIDS [Internet]. SAGE Publications; 2015 Mar 1 [cited 2015 Jun 22];26(4):225–37. Available from: http://std.sagepub.com/content/26/4/225.full |
| 6 | Jespers V, Crucitti T, Menten J, Verhelst R, Mwaura M, Mandaliya K, et al. Prevalence and correlates of bacterial vaginosis in different sub-populations of women in sub-Saharan Africa: a cross-sectional study. PLoS One [Internet]. Public Library of Science; 2014 Jan 7 [cited 2015 May 15];9(10):e109670. Available from: http://journals.plos.org/plosone/article?id=10.1371/journal.pone.0109670 |
| 7 | Guffey MB, Richardson B, Husnik M, Makanani B, Chilongozi D, Yu E, et al. HPTN 035 phase II/IIb randomised safety and effectiveness study of the vaginal microbicides BufferGel and 0.5% PRO 2000 for the prevention of sexually transmitted infections in women. Sex Transm Infect [Internet]. 2014 Aug [cited 2015 Jun 22];90(5):363–9. Available from: http://www.pubmedcentral.nih.gov/articlerender.fcgi?artid=4278566&tool=pmcentrez&rendertype=abstract |
| 8 | Olowe OA, Makanjuola OB, Olowe R, Adekanle DA. Prevalence of vulvovaginal candidiasis, trichomoniasis and bacterial vaginosis among pregnant women receiving antenatal care in Southwestern Nigeria. Eur J Microbiol Immunol (Bp) [Internet]. 2014 Dec [cited 2015 Jun 22];4(4):193–7. Available from: http://www.pubmedcentral.nih.gov/articlerender.fcgi?artid=4271815&tool=pmcentrez&rendertype=abstract |
| 9 | Muvunyi CM, Dhont N, Verhelst R, Temmerman M, Claeys G, Padalko E. Chlamydia trachomatis infection in fertile and subfertile women in Rwanda: prevalence and diagnostic significance of IgG and IgA antibodies testing. Hum Reprod [Internet]. 2011 Dec 1 [cited 2015 Jun 22];26(12):3319–26. Available from: http://humrep.oxfordjournals.org/content/26/12/3319.long |
| 10 | Lazenby GB, Taylor PT, Badman BS, McHaki E, Korte JE, Soper DE, et al. An association between Trichomonas vaginalis and high-risk human papillomavirus in rural Tanzanian women undergoing cervical cancer screening. Clin Ther [Internet]. Elsevier; 2014 Jan 1 [cited 2015 Jun 22];36(1):38–45. Available from: http://www.clinicaltherapeutics.com/article/S0149291813011089/fulltext |
| 11 | McCormack S, Ramjee G, Kamali A, Rees H, Crook AM, Gafos M, et al. PRO2000 vaginal gel for prevention of HIV-1 infection (Microbicides Development Programme 301): a phase 3, randomised, double-blind, parallel-group trial. Lancet [Internet]. Elsevier; 2010 Oct 16 [cited 2015 May 29];376(9749):1329–37. Available from: http://www.thelancet.com/article/S0140673610610860/fulltext |
| 12 | Tchelougou D, Karou DS, Kpotsra A, Balaka A, Assih M, Bamoke M, et al. [Vaginal infections in pregnant women at the Regional Hospital of Sokode (Togo) in 2010 and 2011]. Med Sante Trop [Internet]. Jan [cited 2015 Jun 22];23(1):49–54. Available from: http://www.ncbi.nlm.nih.gov/pubmed/23692693 |
| 13 | Rutherford GW, Anglemyer A, Bagenda D, Muyonga M, Lindan CP, Barker JL, et al. University students and the risk of HIV and other sexually transmitted infections in Uganda: the Crane survey. Int J Adolesc Med Health [Internet]. 2014 Jan [cited 2015 Jun 22];26(2):209–15. Available from: http://www.ncbi.nlm.nih.gov/pubmed/24762640 |
| 14 | Ministry of Health: Botswana. Report on Microbiological Survey of Sexually Transmitted Infections in Botswana, 2007-2008. 2011. Available from: http://www.hiv.gov.bw/sites/default/files/documents/Microbiological%20Survey%20STIs%202007-2008.pdf |
| 15 | Moodley D, Moodley P, Sebitloane M, Soowamber D, McNaughton-Reyes HL, Groves AK, et al. High prevalence and incidence of asymptomatic sexually transmitted infections during pregnancy and postdelivery in KwaZulu Natal, South Africa. Sex Transm Dis [Internet]. 2015 Jan [cited 2015 Jun 22];42(1):43–7. Available from: http://www.ncbi.nlm.nih.gov/pubmed/25504300 |
| 16 | Peters RPH, Dubbink JH, van der Eem L, Verweij SP, Bos MLA, Ouburg S, et al. Cross-sectional study of genital, rectal, and pharyngeal Chlamydia and gonorrhea in women in rural South Africa. Sex Transm Dis [Internet]. 2014 Sep [cited 2015 Jun 22];41(9):564–9. Available from: http://www.ncbi.nlm.nih.gov/pubmed/25118973 |
| 17 | Perazzi BE, Menghi CI, Coppolillo EF, Gatta C, Eliseth MC, de Torres RA, et al. Prevalence and comparison of diagnostic methods for Trichomonas vaginalis infection in pregnant women in Argentina. Korean J Parasitol [Internet]. 2010 Mar [cited 2015 Jun 22];48(1):61–5. Available from: http://www.pubmedcentral.nih.gov/articlerender.fcgi?artid=2843848&tool=pmcentrez&rendertype=abstract |
| 18 | Jalil EM, Pinto VM, Benzaken AS, Ribeiro D, Oliveira EC de, Garcia EG, et al. [Prevalence of Chlamydia and Neisseria gonorrhoeae infections in pregnant women in six Brazilian cities]. Rev Bras Ginecol Obstet [Internet]. 2008 Dec [cited 2015 Jun 22];30(12):614–9. Available from: http://www.ncbi.nlm.nih.gov/pubmed/19219343 |
| 19 | Borborema-Alfaia APB de, Freitas NS de L, Astolfi Filho S, Borborema-Santos CM. Chlamydia trachomatis infection in a sample of northern Brazilian pregnant women: prevalence and prenatal importance. Braz J Infect Dis [Internet]. Jan [cited 2015 Jun 22];17(5):545–50. Available from: http://www.ncbi.nlm.nih.gov/pubmed/23831212 |
| 20 | Gondo DCAF, Duarte MTC, da Silva MG, de Lima Parada CMG. Abnormal vaginal flora in low-risk pregnant women cared for by a public health service: prevalence and association with symptoms and findings from gynecological exams. Rev Lat Am Enfermagem [Internet]. Jan [cited 2015 Jun 22];18(5):919–27. Available from: http://www.ncbi.nlm.nih.gov/pubmed/21120411 |
| 21 | de Lima YAR, Turchi MD, Fonseca ZC, Garcia FLB, de Brito e Cardoso FA, da Guarda Reis MN, et al. Sexually transmitted bacterial infections among young women in Central Western Brazil. Int J Infect Dis [Internet]. Elsevier; 2014 Aug 8 [cited 2015 Jun 22];25:16–21. Available from: http://www.ijidonline.com/article/S1201971214014830/fulltext |
| 22 | Pinto VM, Szwarcwald CL, Baroni C, Stringari LL, Inocêncio LA, Miranda AE. Chlamydia trachomatis prevalence and risk behaviors in parturient women aged 15 to 24 in Brazil. Sex Transm Dis [Internet]. 2011 Oct [cited 2015 Jun 22];38(10):957–61. Available from: http://www.ncbi.nlm.nih.gov/pubmed/21934572 |
| 23 | Mesenburg MA, Stauffert D, Silveira MF. P3.331 Prevalence of Chlamydia Trachomatis Infection and Associated Factors in Brazilian Pregnant Women: Preliminary Results of a Population-Based Study. Sex Transm Infect [Internet]. 2013 Jul 13 [cited 2015 Jun 22];89(Suppl 1):A252–A252. Available from: http://sti.bmj.com/content/89/Suppl_1/A252.3.abstract |
| 24 | Piazzetta RCPS, de Carvalho NS, de Andrade RP, Piazzetta G, Piazzetta SR, Carneiro R. [Prevalence of Chlamydia trachomatis and Neisseria gonorrhoea infections in sexual actives young women at a southern Brazilian city]. Rev Bras Ginecol Obstet [Internet]. 2011 Nov [cited 2015 Jun 22];33(11):328–33. Available from: http://www.ncbi.nlm.nih.gov/pubmed/22267110 |
| 25 | Miranda AE, Pinto VM, Gaydos CA. Trichomonas vaginalis infection among young pregnant women in Brazil. Braz J Infect Dis [Internet]. Jan [cited 2015 Jun 22];18(6):669–71. Available from: http://www.pubmedcentral.nih.gov/articlerender.fcgi?artid=4378618&tool=pmcentrez&rendertype=abstract |
| 26 | Magalhães PAF, Miranda CAN, Lima ÉG, Moizéis RNC, de Lima DBS, Cobucci RNO, et al. Genital tract infection with Chlamydia trachomatis in women attended at a cervical cancer screening program in Northeastern from Brazil. Arch Gynecol Obstet [Internet]. 2015 May [cited 2015 Jun 22];291(5):1095–102. Available from: http://www.ncbi.nlm.nih.gov/pubmed/25326872 |
| 27 | Ovalle A, Martínez MA, de la Fuente F, Falcon N, Feliú F, Fuentealba F, et al. [Prevalence of sexually transmitted infections in pregnant women attending a public hospital in Chile]. Rev Chilena Infectol [Internet]. 2012 Oct [cited 2015 Jun 22];29(5):517–20. Available from: http://www.ncbi.nlm.nih.gov/pubmed/23282493 |
| 28 | López-Monteon A, Gómez-Figueroa FS, Ramos-Poceros G, Guzmán-Gómez D, Ramos-Ligonio A. Codetection of Trichomonas vaginalis and Candida albicans by PCR in urine samples in a low-risk population attended in a clinic first level in central Veracruz, Mexico. Biomed Res Int [Internet]. 2013 Jan [cited 2015 Jun 22];2013:281892. Available from: http://www.pubmedcentral.nih.gov/articlerender.fcgi?artid=3773406&tool=pmcentrez&rendertype=abstract |
| 29 | Cabeza J, García PJ, Segura E, García P, Escudero F, La Rosa S, et al. Feasibility of Chlamydia trachomatis screening and treatment in pregnant women in Lima, Peru: a prospective study in two large urban hospitals. Sex Transm Infect [Internet]. 2015 Feb [cited 2015 Jun 22];91(1):7–10. Available from: http://www.ncbi.nlm.nih.gov/pubmed/25107711 |
| 30 | van der Helm JJ, Bom RJM, Grünberg AW, Bruisten SM, Schim van der Loeff MF, Sabajo LOA, et al. Urogenital Chlamydia trachomatis infections among ethnic groups in Paramaribo, Suriname; determinants and ethnic sexual mixing patterns. PLoS One [Internet]. Public Library of Science; 2013 Jan 17 [cited 2015 Jun 22];8(7):e68698. Available from: http://journals.plos.org/plosone/article?id=10.1371/journal.pone.0068698 |
| 31 | Rashidi BH, Chamani-Tabriz L, Haghollahi F, Jeddi-Tehrani M, Naghizadeh MM, Shariat M, et al. Effects of Chlamydia trachomatis Infection on Fertility; A Case-Control Study. J Reprod Infertil [Internet]. 2013 Apr [cited 2015 Jun 22];14(2):67–72. Available from: http://www.pubmedcentral.nih.gov/articlerender.fcgi?artid=3719322&tool=pmcentrez&rendertype=abstract |
| 32 | Hassanzadeh P, Mardaneh J, Motamedifar M. Conventional Agar-Based Culture Method, and Nucleic Acid Amplification Test (NAAT) of the cppB Gene for Detection of Neisseria gonorrhea in Pregnant Women Endocervical Swab Specimens. Iran Red Crescent Med J [Internet]. 2013 Mar [cited 2015 Jun 22];15(3):207–11. Available from: http://www.pubmedcentral.nih.gov/articlerender.fcgi?artid=3745748&tool=pmcentrez&rendertype=abstract |
| 33 | Marashi SMA, Moulana Z, Imani Fooladi AA, Mashhadi Karim M. Comparison of Genital Chlamydia trachomatis Infection Incidence Between Women With Infertility and Healthy Women in Iran Using PCR and Immunofluorescence Methods. Jundishapur J Microbiol [Internet]. 2014 Apr 1 [cited 2015 Jun 22];7(4):e9450. Available from: http://www.researchgate.net/publication/264991003_Comparison_of_Genital_Chlamydia_trachomatis_Infection_Incidence_Between_Women_With_Infertility_and_Healthy_Women_in_Iran_Using_PCR_and_Immunofluorescence_Methods |
| 34 | Bahram A, Hamid B, Zohre T. Prevalence of Bacterial Vaginosis and Impact of Genital Hygiene Practices in Non-Pregnant Women in Zanjan, Iran. Oman Med J [Internet]. 2009 Oct [cited 2015 Apr 13];24(4):288–93. Available from: http://www.pubmedcentral.nih.gov/articlerender.fcgi?artid=3243866&tool=pmcentrez&rendertype=abstract |
| 35 | Nourian A, Shabani N, Fazaeli A, Mousavinasab SN. Prevalence of Trichomonas vaginalis in Pregnant Women in Zanjan, Northwest of Iran. Jundishapur J Microbiol [Internet]. Kowsar; 2013 Oct 1 [cited 2015 Jun 22];6(8). Available from: http://jjmicrobiol.com/7258.abstract |
| 36 | Arbabi M, Fakhrieh Z, Delavari M, Abdoli A. Prevalence of Trichomonas vaginalis infection in Kashan city, Iran (2012-2013). Iran J Reprod Med [Internet]. 2014 Jul [cited 2015 Jun 22];12(7):507–12. Available from: http://www.pubmedcentral.nih.gov/articlerender.fcgi?artid=4126256&tool=pmcentrez&rendertype=abstract |
| 37 | Al-Sweih NA, Khan S, Rotimi VO. Prevalence of Chlamydia trachomatis and Neisseria gonorrhoeae among asymptomatic women attending the Capital Health region clinics in Kuwait. Sex Transm Dis [Internet]. 2011 Sep [cited 2015 May 15];38(9):793–7. Available from: http://www.ncbi.nlm.nih.gov/pubmed/21844731 |
| 38 | Ministry of Health: Morocco. National AIDS Program. 2014. Data provided by A Hancali in September 2014 |
| 39 | Al-Thani A, Abdul-Rahim H, Alabsi E, Bsaisu HN, Haddad P, Mumtaz GR, et al. Prevalence of Chlamydia trachomatis infection in the general population of women in Qatar. Sex Transm Infect [Internet]. 2013 Nov [cited 2015 Jun 22];89 Suppl 3:iii57–60. Available from: http://www.pubmedcentral.nih.gov/articlerender.fcgi?artid=3841785&tool=pmcentrez&rendertype=abstract |
| 40 | Munro HL, Pradeep BS, Jayachandran AA, Lowndes CM, Mahapatra B, Ramesh BM, et al. Prevalence and determinants of HIV and sexually transmitted infections in a general population-based sample in Mysore district, Karnataka state, southern India. AIDS [Internet]. 2008 Dec [cited 2015 Jun 22];22 Suppl 5:S117–25. Available from: http://www.ncbi.nlm.nih.gov/pubmed/19098472 |
| 41 | Rajaram S, Sangameshwar BM, Jayachandran AA, Bradley J, Alary M, Ramesh B, et al. HIV and STIs in Bellary District, Karnataka, India. A general population survey. 2009. Report No.: 3. |
| 42 | Rajaram S, Sangameshwar BM, Jayachandran AA, Badley J, Alary M, Ramesh B, et al. HIV and STIs in Belgaum District, Karnataka, India. A general population survey. 2008. Report No.: 1. |
| 43 | Vidwan NK, Regi A, Steinhoff M, Huppert JS, Staat MA, Dodd C, et al. Low prevalence of Chlamydia trachomatis infection in non-urban pregnant women in Vellore, S. India. PLoS One [Internet]. Public Library of Science; 2012 Jan 2 [cited 2015 Jun 22];7(5):e34794. Available from: http://journals.plos.org/plosone/article?id=10.1371/journal.pone.0034794 |
| 44 | Lu Q, Yuan C, Xie C, Al E. Analysis on the detection results in 11254 cases of urogenital Chlamydia trachomatis infection by fluorescent PCR. Chinese J AIDS STDs. 2013;19(10):760–6. |
| 45 | Caiyan X, Weiyuan Z, Minghui W, Songwen Z. Prevalence and risk factors of lower genital tract infections among women in Beijing, China. J Obstet Gynaecol Res [Internet]. 2012 Jan [cited 2015 Jun 22];38(1):310–5. Available from: http://www.ncbi.nlm.nih.gov/pubmed/21827575 |
| 46 | Dai Q, Hu L, Jiang Y, Shi H, Liu J, Zhou W, et al. An epidemiological survey of bacterial vaginosis, vulvovaginal candidiasis and trichomoniasis in the Tibetan area of Sichuan Province, China. Eur J Obstet Gynecol Reprod Biol [Internet]. Elsevier; 2010 Jun 6 [cited 2015 Jun 22];150(2):207–9. Available from: http://www.ejog.org/article/S030121151000093X/fulltext |
| 47 | Fang X, Zhou Y, Yang Y, Diao Y, Li H. Prevalence and risk factors of trichomoniasis, bacterial vaginosis, and candidiasis for married women of child-bearing age in rural Shandong. Jpn J Infect Dis [Internet]. 2007 Sep [cited 2015 Jun 22];60(5):257–61. Available from: http://www.ncbi.nlm.nih.gov/pubmed/17881863 |
| 48 | Wen CH et al. Research on the association between HPV infection and other common genital infections. Maternal and Child Health Care of China. 2013; 28(24):3985-8. CNKI link (not full text):http://www.cnki.net/KCMS/detail/detail.aspx?QueryID=75&CurRec=4&dbcode=CJFQ&dbname=CJFD2013&filename=ZFYB201324042&urlid=&yx=&v=MjI0ODNVUkwrZlllZHFGeTNnVWJ2TVB5dlNiTEc0SDlMT3E0OUJab1I4ZVgxTHV4WVM3RGgxVDNxVHJXTTFGckM= |
| 49 | Zhang X-J, Shen Q, Wang G-Y, Yu Y-L, Sun Y-H, Yu G-B, et al. Risk factors for reproductive tract infections among married women in rural areas of Anhui Province, China. Eur J Obstet Gynecol Reprod Biol [Internet]. Elsevier; 2009 Dec 12 [cited 2015 Jun 22];147(2):187–91. Available from: http://www.ejog.org/article/S0301211509005065/fulltext |
| 50 | Saison F, Mahilum-Tapay L, Michel C-EE, Buttress ND, Nadala ECB, Magbanua JP V, et al. Prevalence of Chlamydia trachomatis infection among low- and high-risk Filipino women and performance of Chlamydia rapid tests in resource-limited settings. J Clin Microbiol [Internet]. 2007 Dec [cited 2015 Jun 22];45(12):4011–7. Available from: http://www.pubmedcentral.nih.gov/articlerender.fcgi?artid=2168544&tool=pmcentrez&rendertype=abstract |
| 51 | Herath S, Samarakoon S, Fonseka P. Epidemiology of reproductive tract infections (RTI) among married reproductive aged women in low socioeconomic communities of Sri Lanka: a country with low level HIV epidemic. AIDS 2010 - XVIII Int AIDS Conf [Internet]. 2010 [cited 2015 Jun 23];Abstract no. MOPEO180. Available from: <https://www.aids2014.org/Abstracts/A200735406.aspx>. Additional details provided by lead author |
| 52 | Lan PT, Lundborg CS, Phuc HD, Sihavong A, Unemo M, Chuc NTK, et al. Reproductive tract infections including sexually transmitted infections: a population-based study of women of reproductive age in a rural district of Vietnam. Sex Transm Infect [Internet]. 2008 Apr 1 [cited 2015 Jun 22];84(2):126–32. Available from: http://sti.bmj.com/content/84/2/126.short |
| 53 | Chen MY, Fairley CK, De Guingand D, Hocking J, Tabrizi S, Wallace EM, et al. Screening pregnant women for chlamydia: what are the predictors of infection? Sex Transm Infect [Internet]. 2009 Feb [cited 2015 Jun 22];85(1):31–5. Available from: http://www.ncbi.nlm.nih.gov/pubmed/18708481 |
| 54 | Walker J, Fairley CK, Bradshaw CS, Tabrizi SN, Chen MY, Twin J, et al. “The difference in determinants of Chlamydia trachomatis and Mycoplasma genitalium in a sample of young Australian women”. BMC Infect Dis [Internet]. 2011 Jan [cited 2015 Jun 22];11(1):35. Available from: http://www.biomedcentral.com/1471-2334/11/35 |
| 55 | Yeung AH, Temple-Smith M, Fairley CK, Vaisey AM, Guy R, Law MG, et al. Chlamydia prevalence in young attenders of rural and regional primary care services in Australia: a cross-sectional survey. Med J Aust [Internet]. 2014 Feb 17 [cited 2015 Jun 22];200(3):170–5. Available from: http://www.ncbi.nlm.nih.gov/pubmed/24528434 |
| 56 | Imai H, Nakao H, Shinohara H, Fujii Y, Tsukino H, Hamasuna R, et al. Population-based study of asymptomatic infection with Chlamydia trachomatis among female and male students. Int J STD AIDS [Internet]. 2010 May [cited 2015 Jun 22];21(5):362–6. Available from: http://www.ncbi.nlm.nih.gov/pubmed/20498109 |
| 57 | Yamazaki T, Matsumoto M, Matsuo J, Abe K, Minami K, Yamaguchi H. Frequency of Chlamydia trachomatis in Ureaplasma-positive healthy women attending their first prenatal visit in a community hospital in Sapporo, Japan. BMC Infect Dis [Internet]. 2012 Jan [cited 2015 Jun 22];12(1):82. Available from: http://www.biomedcentral.com/1471-2334/12/82 |
| 58 | Choe H-S, Lee DS, Lee S-J, Lee CB, Lee WC, Cho Y-H. Prevalence of sexually transmitted infections and sexual behavior of young adults and middle-aged people presenting to health examination centers in Korea. J Infect Chemother [Internet]. 2012 Apr [cited 2015 Jun 22];18(2):207–12. Available from: http://www.ncbi.nlm.nih.gov/pubmed/21989518 |
| 59 | Hahn H-S, Lee K-H, Koo Y-J, Kim S-G, Rhee JE, Kim MY, et al. Distribution and perinatal transmission of bacterial vaginal infections in pregnant women without vaginal symptoms. Scand J Infect Dis [Internet]. Informa Healthcare Stockholm; 2014 May 15 [cited 2015 Jun 22];46(5):348–53. Available from: http://informahealthcare.com/doi/abs/10.3109/00365548.2014.880183 |
| 60 | Kim S-J, Lee DS, Lee S-J. The prevalence and clinical significance of urethritis and cervicitis in asymptomatic people by use of multiplex polymerase chain reaction. Korean J Urol [Internet]. 2011 Oct [cited 2015 Jun 22];52(10):703–8. Available from: http://www.pubmedcentral.nih.gov/articlerender.fcgi?artid=3212666&tool=pmcentrez&rendertype=abstract |
| 61 | World Health Organization. Regional Office for the Western Pacific. Second Generation Surveillance Surveys of HIV, other STIs and Risk Behaviours in 6 Pacific Island Countries (2004-2005) [Internet]. WPRO \| WHO Western Pacific Region; 2006 [cited 2015 Jun 23]. Available from: http://www.wpro.who.int/hiv/documents/SGS_FINAL_DOCUMENT/en/ |
| 62 | Wangnapi RA, Soso S, Unger HW, Sawera C, Ome M, Umbers AJ, et al. Prevalence and risk factors for Chlamydia trachomatis, Neisseria gonorrhoeae and Trichomonas vaginalis infection in pregnant women in Papua New Guinea. Sex Transm Infect [Internet]. 2015 May [cited 2015 Jun 22];91(3):194–200. Available from: http://www.ncbi.nlm.nih.gov/pubmed/25313204 |
| 63 | Ministry of Health: Government of Tonga, Secretariat of the Pacific Community. Second generation HIV surveillance in antenatal clinic attendees and youth, Tonga, 2008. 2008. Available from: http://www.spc.int/hiv/index2.php?option=com_docman&task=doc_view&gid=210&Itemid=148 |
| 64 | Homasi S. HIV/AIDS and other STIs in Tuvalu South Pacific. MMedicine thesis. University of Sydney. August 2007. |
| 65 | Ministry of Health: Vanuatu, Secretariat of the Pacific Community. Second generation surveillance of antenatal women, STI clinic clients and youth, Vanuatu, 2008. 2008. Available from: http://www.spc.int/hiv/index2.php?option=com_docman&task=doc_view&gid=246&Itemid=1 |
| 66 | Secretariat of the Pacific Community. Second generation surveys on HIV and High-risk behaviour in New Caledonia. 2007. |
| 67 | Ministry of Health: Mongolia. STI prevalence survey among antenatal attendees, 2008. 2008. |
| 68 | European Centre for Disease Control 2008 == ANRS.INED.INSERM.. Dossier de presse. Premiers resultats de le'enquete CSF "Contexte de la sexulaite en France". 13-3-2007. ANRS. INED. INSERM. Quoted in ECDC Technical Report: Review of Clamydia Control Activities in EU Countries. May 2008 |
| 69 | Mossong J, Muller M, Majéry N, Mardaga C, Decruyenaere F, Schneider F. Screening for Chlamydia trachomatis in secondary schools, family planning and occupational health centres in Luxembourg. Sex Transm Infect [Internet]. 2009 Oct [cited 2015 Jun 22];85(6):455–8. Available from: http://www.ncbi.nlm.nih.gov/pubmed/19574242 |
| 70 | Fernández-Benítez C, Mejuto-López P, Otero-Guerra L, Margolles-Martins MJ, Suárez-Leiva P, Vazquez F. Prevalence of genital Chlamydia trachomatis infection among young men and women in Spain. BMC Infect Dis [Internet]. 2013 Jan [cited 2015 Jun 22];13(1):388. Available from: http://www.biomedcentral.com/1471-2334/13/388 |
| 71 | Sonnenberg P, Clifton S, Beddows S, Field N, Soldan K, Tanton C, et al. Prevalence, risk factors, and uptake of interventions for sexually transmitted infections in Britain: findings from the National Surveys of Sexual Attitudes and Lifestyles (Natsal). Lancet [Internet]. Elsevier; 2013 Nov 30 [cited 2015 May 25];382(9907):1795–806. Available from: http://www.thelancet.com/article/S0140673613619479/fulltext |
| 72 | Torrone E, Papp J, Weinstock H. Prevalence of Chlamydia trachomatis genital infection among persons aged 14-39 years--United States, 2007-2012. MMWR Morb Mortal Wkly Rep [Internet]. 2014 Sep 26 [cited 2015 Jun 22];63(38):834–8. Available from: http://www.ncbi.nlm.nih.gov/pubmed/25254560 |
| 73 | Satterwhite CL, Torrone E, Meites E, Dunne EF, Mahajan R, Ocfemia MCB, et al. Sexually transmitted infections among US women and men: prevalence and incidence estimates, 2008. Sex Transm Dis [Internet]. 2013 Mar [cited 2015 Jun 22];40(3):187–93. Available from: http://www.ncbi.nlm.nih.gov/pubmed/23403598 |
| 74 | Brasil. Ministério da Saúde. Secretaria de Vigilância em Saúde. Programa Nacional de DST e Aids. Prevalências e freqüências relativas de Doenças Sexualmente Transmissíveis (DST) em populações selecionadas de seis capitais brasileiras, 2005. 2008. Available from: http://www.hiv.gov.bw/sites/default/files/documents/Microbiological%20Survey%20STIs%202007-2008.pdf |
| 75 | Alberts CJ, Schim van der Loeff MF, Papenfuss MR, da Silva RJC, Villa LL, Lazcano-Ponce E, et al. Association of Chlamydia trachomatis infection and herpes simplex virus type 2 serostatus with genital human papillomavirus infection in men: the HPV in men study. Sex Transm Dis [Internet]. 2013 Jun [cited 2015 Jun 22];40(6):508–15. Available from: http://www.pubmedcentral.nih.gov/articlerender.fcgi?artid=3904659&tool=pmcentrez&rendertype=abstract |
| 76 | Meidani M. Frequency of chlamydia trachomatis infection in asymptomatic males based on PCR in Tehran, Iran. Int Urogynecol J Pelvic Floor Dysfunct. 2009;20(Supplement 3):S460. |
| 77 | Mir AM, Wajid A, Reichenbach L, Khan M. STI prevalence and associated factors among urban men in Pakistan. Sex Transm Infect [Internet]. 2009 Jun [cited 2015 Jun 22];85(3):199–200. Available from: http://www.ncbi.nlm.nih.gov/pubmed/19211591 |
| 78 | Jatapai A, Sirivongrangson P, Lokpichat S, Chuenchitra T, Nelson KE, Rangsin R. Prevalence and risk factors for Chlamydia trachomatis infection among young Thai men in 2008-2009. Sex Transm Dis [Internet]. 2013 Mar [cited 2015 Jun 22];40(3):241–6. Available from: http://www.ncbi.nlm.nih.gov/pubmed/23407469 |
| 79 | Baud D, Jaton K, Bertelli C, Kulling J-P, Greub G. Low prevalence of Chlamydia trachomatis infection in asymptomatic young Swiss men. BMC Infect Dis [Internet]. 2008 Jan [cited 2015 Jun 22];8(1):45. Available from: http://www.biomedcentral.com/1471-2334/8/45 |
