# Supplementary material for: Global Estimates of the Prevalence and Incidence of Four Curable Sexually Transmitted Infections in 2012 Based on Systematic Review and Global Reporting
Source: PLoS One. 2015 Dec 8;10(12):e0143304. doi: 10.1371/journal.pone.0143304 (PMC4672879; doi:10.1371/journal.pone.0143304)
Supplement: S1 Tables — (DOCX) [file pone.0143304.s005.docx]

**S1 Tables:**

**Global and regional estimates for 2012 by infection and sex of the percentage of population with prevalent infection and the estimated incidence rates (per 1,000)**

| **Table A. Percentage of population (%) with prevalent infection in 2012 by infection and sex (95% uncertainty interval shown in parentheses)** | | | | |
| --- | --- | --- | --- | --- |
|  | **Women** | | | |
| **Region** | **Chlamydia** | **Gonorrhoea** | **Trichomoniasis** | **Syphilis** |
| African Region | 3.7 ( 2.7 - 5.2 ) | 1.7 ( 1.1 - 2.6 ) | 11.5 ( 9.0 - 14.6 ) | 1.77 ( 1.41 - 2.52 ) |
| Region of the Americas | 7.6 ( 6.7 - 8.7 ) | 0.8 ( 0.5 - 1.1 ) | 7.7 ( 4.3 - 13.1 ) | 0.41 ( 0.36 - 0.50 ) |
| Eastern Mediterranean Region | 3.5 ( 2.4 - 5.0 ) | 0.5 ( 0.3 - 0.7 ) | 5.9 ( 4.5 - 8.0 ) | 0.50 ( 0.37 - 0.86 ) |
| European Region | 2.2 ( 1.6 - 2.9 ) | 0.3 ( 0.2 - 0.5 ) | 1.0 ( 0.8 - 1.3 ) | 0.17 ( 0.07 - 0.41 ) |
| South-East Asia Region | 1.8 ( 1.4 - 2.2 ) | 0.4 ( 0.2 - 0.5 ) | 1.8 ( 1.1 - 2.7 ) | 0.37 ( 0.33 - 0.42 ) |
| Western Pacific Region | 6.2 ( 5.1 - 7.5 ) | 1.2 ( 0.8 - 1.7 ) | 5.5 ( 3.3 - 8.9 ) | 0.23 ( 0.21 - 0.25 ) |
| Global total (women) | 4.2 ( 3.7 - 4.7 ) | 0.8 ( 0.6 - 1.0 ) | 5.0 ( 4.0 - 6.4 ) | 0.49 ( 0.43 - 0.59 ) |
|  | **Men** | | | |
| **Region** | **Chlamydia** | **Gonorrhoea** | **Trichomoniasis** | **Syphilis** |
| African Region | 2.5 ( 1.7 - 3.6 ) | 0.5 ( 0.3 - 0.9 ) | 1.2 ( 0.7 - 1.7 ) | 1.76 ( 1.07 - 2.81 ) |
| Region of the Americas | 1.8 ( 1.3 - 2.6 ) | 0.7 ( 0.4 - 1.0 ) | 1.3 ( 0.9 - 2.0 ) | 0.41 ( 0.27 - 0.58 ) |
| Eastern Mediterranean Region | 2.7 ( 1.6 - 4.3 ) | 0.4 ( 0.2 - 0.6 ) | 0.6 ( 0.4 - 0.9 ) | 0.48 ( 0.27 - 0.88 ) |
| European Region | 1.5 ( 0.9 - 2.6 ) | 0.3 ( 0.2 - 0.5 ) | 0.1 ( 0.1 - 0.2 ) | 0.16 ( 0.06 - 0.42 ) |
| South-East Asia Region | 1.3 ( 0.9 - 1.8 ) | 0.5 ( 0.3 - 0.8 ) | 0.2 ( 0.1 - 0.3 ) | 0.37 ( 0.24 - 0.50 ) |
| Western Pacific Region | 5.2 ( 3.5 - 7.2 ) | 1.0 ( 0.6 - 1.7 ) | 0.6 ( 0.3 - 1.0 ) | 0.23 ( 0.15 - 0.30 ) |
| Global total (men) | 2.7 ( 2.0 - 3.6 ) | 0.6 ( 0.4 - 0.9 ) | 0.6 ( 0.4 - 0.8 ) | 0.48 ( 0.31 - 0.67 ) |

| **Table B. Incidence rates (per 1,000) by infection and sex, based on 2005 to 2012 data (95% uncertainty interval shown in parentheses).** | | | | |
| --- | --- | --- | --- | --- |
|  | **Women** | | | |
| **Region** | **Chlamydia** | **Gonorrhoea** | **Trichomoniasis** | **Syphilis** |
| African Region | 31 ( 19 - 49 ) | 37 ( 20 - 62 ) | 83 ( 53 - 122 ) | 4.4 ( 2.7 - 6.8 ) |
| Region of the Americas | 72 ( 50 - 96 ) | 19 ( 10 - 30 ) | 56 ( 26 - 107 ) | 1.9 ( 1.3 - 2.8 ) |
| Eastern Mediterranean Region | 31 ( 19 - 49 ) | 12 ( 7 - 18 ) | 47 ( 30 - 69 ) | 1.5 ( 0.9 - 3.1 ) |
| European Region | 21 ( 13 - 30 ) | 8 ( 5 - 14 ) | 8 ( 5 - 12 ) | 1.0 ( 0.4 - 2.2 ) |
| South-East Asia Region | 15 ( 10 - 21 ) | 8 ( 4 - 13 ) | 13 ( 7 - 21 ) | 0.9 ( 0.6 - 1.2 ) |
| Western Pacific Region | 56 ( 39 - 76 ) | 29 ( 16 - 46 ) | 44 ( 24 - 74 ) | 1.0 ( 0.7 - 1.3 ) |
| Global total (women) | **38 ( 28 - 48 )** | **19 ( 13 - 26 )** | **38 ( 26 - 53 )** | **1.5 ( 1.1 - 2.0 )** |
|  | **Men** | | | |
| **Region** | **Chlamydia** | **Gonorrhoea** | **Trichomoniasis** | **Syphilis** |
| African Region | 26 ( 15 - 40 ) | 17 ( 9 - 30 ) | 94 ( 49 - 156 ) | 4.4 ( 2.3 - 7.5 ) |
| Region of the Americas | 29 ( 18 - 42 ) | 26 ( 13 - 46 ) | 56 ( 23 - 113 ) | 1.9 ( 1.0 - 3.2 ) |
| Eastern Mediterranean Region | 33 ( 17 - 58 ) | 16 ( 8 - 26 ) | 48 ( 26 - 81 ) | 1.5 ( 0.7 - 3.1 ) |
| European Region | 20 ( 10 - 36 ) | 13 ( 6 - 22 ) | 9 ( 5 - 15 ) | 1.0 ( 0.4 - 2.4 ) |
| South-East Asia Region | 13 ( 7 - 21 ) | 15 ( 8 - 26 ) | 14 ( 7 - 25 ) | 0.9 ( 0.5 - 1.4 ) |
| Western Pacific Region | 64 ( 39 - 98 ) | 41 ( 19 - 72 ) | 46 ( 21 - 85 ) | 1.0 ( 0.5 - 1.5 ) |
| Global total (men) | **33 ( 22 - 47 )** | **24 ( 14 - 37 )** | **40 ( 22 - 63 )** | **1.5 ( 0.9 - 2.3 )** |
