# Supplementary material for: Global Estimates of the Prevalence and Incidence of Four Curable Sexually Transmitted Infections in 2012 Based on Systematic Review and Global Reporting
Source: PLoS One. 2015 Dec 8;10(12):e0143304. doi: 10.1371/journal.pone.0143304 (PMC4672879; doi:10.1371/journal.pone.0143304)
Supplement: S1 Text — (DOCX) [file pone.0143304.s006.docx]

**S1 Text: Search strategy**

**Search Strategy**

The PubMed searches were conducted for individual countries using the following terms: "chlamydia"[MeSH Terms] OR "chlamydia"[All Fields], "gonorrhoea"[All Fields] OR "gonorrhea"[MeSH Terms] OR "gonorrhea"[All Fields], "trichomonas infections"[MeSH Terms] OR ("trichomonas"[All Fields] AND "infections"[All Fields]) OR "trichomonas infections"[All Fields] OR "trichomoniasis"[All Fields], with a filter of publication date from 01/01/05 to 30/01/15.

**Study inclusion criteria**

- Sample size of at least 100;
- Specimens collected from 2005 through 2012. For studies in which no specimen collection date was specified, the study had to be published in 2006 or later;
- Population could be considered representative of the general population (e.g., studies conducted in pregnant women, women at delivery, women attending family planning clinics, military recruits, or individuals selected for participation in a Demographic and Health Survey (DHS));
- No apparent bias in the selection of study participants (e.g., studies conducted among the following groups were excluded: patients seeking care for an STI or genital symptoms, women with abnormal Papanicolaou (pap) smears, blood donors, women attending gynecology or sexual health clinics, and remote or indigenous populations); and
- Used an internationally recognized diagnostic test with adequate performance characteristics on urine, urethral, or cervicovaginal specimens.
