# Supplementary material for: Global Estimates of the Prevalence and Incidence of Four Curable Sexually Transmitted Infections in 2012 Based on Systematic Review and Global Reporting
Source: PLoS One. 2015 Dec 8;10(12):e0143304. doi: 10.1371/journal.pone.0143304 (PMC4672879; doi:10.1371/journal.pone.0143304)
Supplement: S3 Text — (DOCX) [file pone.0143304.s008.docx]

**S3 Text: Standardizing prevalence data across studies**

The methods for standardizing data from prevalence studies for the 2012 estimates were based on the methods used by WHO in 2005 and 2008 [6]. A standardized prevalence data point was obtained by adjusting the reported prevalence to take into account the sensitivity and specificity of the diagnostic test used, the age of the study population (for chlamydia only), and the geographic location of the study population using the following formulas:

SP = (RP + Specificity – 1)

________________ × age adjustor × geography adjustor

(Sensitivity + Specificity – 1)

where SP is the standardized prevalence, RP is the reported prevalence, specificity is the reported specificity of the lab test and sensitivity is the reported sensitivity of the lab test. The age and geography adjustors are described below.

When the equation was applied to the data and a negative number was generated for the standardized prevalence the standardized prevalence was estimated to be 0.1.

**Laboratory test adjustors**

The performance characteristics of each laboratory test type were based on the most recent systematic review, and were the same values as used for 2005 and 2008 estimates (Table A).

**Table A. Sensitivity and specificity of laboratory tests**

| **Sex** | **Specimen** | **Test** | **Sensitivity %** | **Specificity %** | |
| --- | --- | --- | --- | --- | --- |
| **Chlamydia** | | | | | |
| Women | Genital fluid | NAAT | 88.6 | 99.6 | |
|  |  | Culture | 63.4 | 100 | |
|  |  | ELISA | 65 | 100 | |
|  | Urine | NAAT | 87.0 | 99.8 | |
| Men | Genital fluid | NAAT | 87.5 | 99.2 | |
|  | Urine | NAAT | 87.8 | 99.3 | |
| **Gonorrhoea** | | | | | |
| Women | Genital fluid | NAAT | 93.3 | 99.2 | |
|  |  | Culture | 75.7 | 100 | |
|  | Urine | NAAT | 91.6 | 100 | |
| Men | Genital fluid | NAAT | 96.1 | 99.0 | |
|  |  | Culture | 87.6 | 100 | |
|  | Urine | NAAT | 80.9 | 99.9 | |
| **Trichomoniasis** | | | | | |
| Women | Genital fluid | NAAT | 95.0 | | 98.0 |
|  |  | Culture | 68.8 | | 100 |
|  |  | MWM | 52 | | 100 |
|  | Urine | NAAT | 66.9 | | 98.3 |
| Men | Genital fluid | NAAT | 81.6 | | 97.7 |
|  |  | MWM | 44.0 | | 100 |
|  |  | Culture | 74.5 | | 100 |
|  | Urine | NAAT | 96.0 | | 97.7 |
| *ELISA, enzyme linked immunosorbent assay; MWM, manual wet mount; NAAT, nucleic acid amplification test.* | | | | | |

**Age adjustor:**

Prevalence data for chlamydia in women were systematically adjusted for age, but no adjustments were made for the other three infections because although prevalence may vary by age, there were insufficient data to generate credible weights. The decision to adjust the prevalence data for women for chlamydia was based on the observed variation in the prevalence of chlamydia among women of different ages. For example, Franceschi et al*.* conducted an investigation across four continents that revealed greater prevalence in women aged 15–24 years than in those aged 25–44 years; chlamydia infected 4.5% vs. 2.6% in these age groups respectively (the population prevalence was 3.0%). ^[[1]](#endnote-1)^In contrast, they showed that gonorrhoea had similar prevalence in both age groups. Table B records the age adjustors used.

Table B. Age adjustors for estimates for women with chlamydial infection

| **Age range (years)** | **Adjustment factor** |
| --- | --- |
| Youths (15–24) | 0.67 |
| Adults (25–49) | 1.15 |
| Other (or no) information | 1.0 |

**Geography adjustor:**

For the 2005 estimates, a review was conducted of studies where information was available for both rural and urban areas. This review found that the prevalence in urban areas was generally higher than in rural areas. The number of studies with both urban and rural data was very small and were primarily studies looking at syphilis. Drawing on these data and the 2008 WHO Expert Consultation the ratio of the prevalence in rural to urban areas for all four infections in all regions of the world was estimated to be 0.9.

The value of the geography adjustor was based on the ratio of the prevalence of the infection in rural areas to urban areas as well as estimates of the proportion of the population living in urban areas, using the UN World Urbanization Prospects: 2011 Revision data (United Nations, 2012) for less developed (Africa, Asia (excluding Japan), Latin America and the Caribbean, Melanesia, Federated States of Micronesia and Polynesia) and more developed regions. This report estimated that 46.5% of people lived in urban areas in less developed regions and 77.7% in more developed regions. No geography adjustor was used for High Income North America. Table C records the geography adjustors used.

Table C. Geography adjustors

| **Type of population** | **Adjustor for all regions except WHO European Region** | **Adjustor for WHO European Region** |
| --- | --- | --- |
| National | 1 | 1 |
| Urban | 0.947 | 0.978 |
| Rural | 1.052 | 1.086 |
| Unknown | 1 | 1 |

1. Franceschi S, Smith JS, van den Brule A, Herrero R, Arslan A, Anh PT, et al. Cervical infection with *Chlamydia trachomatis* and *Neisseria gonorrhoeae* in women from ten areas in four continents: a cross-sectional study. Sex Transm Dis. 2007;34:563–569. [↑](#endnote-ref-1)
