# Supplementary material for: Global Estimates of the Prevalence and Incidence of Four Curable Sexually Transmitted Infections in 2012 Based on Systematic Review and Global Reporting
Source: PLoS One. 2015 Dec 8;10(12):e0143304. doi: 10.1371/journal.pone.0143304 (PMC4672879; doi:10.1371/journal.pone.0143304)
Supplement: S4 Text — (DOCX) [file pone.0143304.s009.docx]

**S4 Text: Estimating duration of infection**

The duration of an infection, or the mean length of time that a person carries an infection within a population, depends on a number of factors. These include the average duration of infection in the absence of treatment for both people who are symptomatic and asymptomatic, and treatment patterns for those who are symptomatic within a population. In addition, people who are asymptomatic may receive treatment because of screening programmes, or partner notification, or inadvertent correct therapy while being treated for another health problem. The methods used to estimate the mean duration of each infection were the same in 2005 and 2008 as in 1995 and 1999, although some changes were made to the numerical values assigned to certain parameters [5-8].

###### For chlamydia, gonorrhoea and trichomoniasis, the duration of infection (D) for a person of sex (k) in treatment area (r) was estimated using the following equation:

*D*(*k,r*) = *S*(*k,r*) x [*V*^S^(*k,r*) × *T*^S^(*k,r*) + (1 – *V*^S^(*k,r*)) × *U*^S^(*k,r*)]
+ (1 – *S*(*k,r*))
× [*V*^A^(*k,r*) × *T*^A^(*k,r*) + (1 – *V*^A^(*k,r*)) × *U*^A^(*k,r*)]

where:

S(*k*,*r*) was the probability that an infected person is symptomatic (S).

*V*^S^(*k*,*r*) and *V*^A^(*k*,*r*) was the probabilities that infected people who were *symptomatic* (*S*) and *asymptomatic* (*A*) were treated, respectively.

*T*^S^(*k*,*r*) and *U*^S^(*k*,*r*) was the average durations of infections for symptomatic (*S*) people who were treated (*T*) and not treated (*U*), respectively.

*T*^A^(*k*,*r*) and *U*^A^(*k*,*r*) was the average durations of infections for asymptomatic (A) people who were treated and not treated, respectively.

**Adjusting for access to treatment:** Treatment patterns vary widely between countries and within countries, and this situation depends on access to healthcare as well as cultural and economic factors. To adjust for these factors, the same approach was applied in 2012 as was used in 2005 and 2008. The 10 regions were divided into one of three treatment groups based on the probability of someone with an infection being appropriately treated (Table A4.1). Western Europe was separated from Central and Eastern Europe, and Central Asia.

**Table A4.1 Allocation of regions into one of three treatment groups according to probability of treatment**

| **Treatment group** | **Region for 2012 estimates** | **WHO region – 2008 estimates** |
| --- | --- | --- |
| A | - Australasia and High Income Asia Pacific - High income North America - Western Europe | WHO European Region  North America (Canada and USA) |
| B | - Andean, Central, Southern, Tropical Latin America & Caribbean - North Africa & Middle East - Oceania - East Asia - Central & Eastern Europe & Central Asia | WHO Eastern Mediterranean Region  WHO Region of the Americas (excluding North America) WHO Western Pacific Region |
| C | - Central, Eastern & Western Sub-Saharan Africa - Southern Sub-Saharan Africa - South Asia & South East Asia | WHO African Region,  WHO South-East Asia Region |

**Parameter values:** The values assigned to the parameters used to generate estimates of duration were reviewed in preparation for the 2012 estimates and it was decided to use the same values as those used to generate the 2008 estimates (Tables A4.2–A4.4) which were based on a literature review updated in 2008.

**Table A4.2 Probability of men and women developing symptoms**

| **Infection** | **Men** | **Women** |
| --- | --- | --- |
| Chlamydia | 0.54 | 0.17 |
| Gonorrhoea | 0.64 | 0.34 |
| Trichomoniasis | 0.067 | 0.34 |

**Table A4.3 Average duration of infection**

| **Infection** | **Asymptomatic and not treated** | | **Symptomatic and treated** | |
| --- | --- | --- | --- | --- |
|  | **Men** | **Women** | **Men** | **Women** |
| Chlamydia | 15 months | 15 months | 4 weeks | 8 weeks |
| Gonorrhoea | 5 months | 6 months | 2 weeks | 4 weeks |
| Trichomoniasis | 1.5 months | 18 months | 1 month | 3 months |

**Table A4.4 Probability that a symptomatic person was adequately treated by treatment group^†^**

| **Treatment group** | **Probability in men** | **Probability in women** |
| --- | --- | --- |
| A | 0.80 | 0.75 |
| B | 0.65 | 0.50 |
| C | 0.35 | 0.225 |

*^†^The probability that an asymptomatic person is treated was assumed to be 10% of the probability that a symptomatic person is treated.*

A similar approach was followed for syphilis, after adjusting for the different stages of infection (primary, secondary, and latent) (Tables A4.5–A4.6). The probability of adequate treatment was assumed to be the same for men and women. It was also assumed that people with primary and secondary syphilis who did not develop symptoms were not treated.

**Table A4.5 Average duration of infection in a person with syphilis depending on stage at which they are treated**

| ***Stage of infection*** | ***Probability*** |
| --- | --- |
| *Primary* | *1 month* |
| *Secondary* | *3 months* |
| *Latent* | *3 years* |
| *Tertiary* | *15 years* |

**Table A4.6 Probability that a person is adequately treated for syphilis according to treatment group (see table A4.1 for how groups are allocated)**

| **Treatment group** | **Symptomatic primary and secondary stages** | **Latent stages** |
| --- | --- | --- |
| A | 0.85 | 0.95 |
| B | 0.60 | 0.85 |
| C | 0.35 | 0.75 |

***Duration of infection values:*** The average duration of the four infections in each of the four treatment areas is presented in Table A4.7.

**Table A4.7 Average duration of infection by sex and treatment group**

| **Infection** | **Treatment Group** | **Duration of infection (years)** | |
| --- | --- | --- | --- |
|  |  | **Men** | **Women** |
| Chlamydia | A | 0.7 | 1.04 |
|  | B | 0.8 | 1.11 |
|  | C | 1.01 | 1.19 |
| Gonorrhoea | A | 0.21 | 0.37 |
|  | B | 0.25 | 0.41 |
|  | C | 0.33 | 0.46 |
| Trichomoniasis | A | 0.12 | 1.12 |
|  | B | 0.12 | 1.25 |
|  | C | 0.12 | 1.39 |
| Syphilis | A | 1.28 | 1.28 |
|  | B | 2.42 | 2.42 |
|  | C | 4.13 | 4.13 |
