# Supplementary material for: Global Estimates of the Prevalence and Incidence of Four Curable Sexually Transmitted Infections in 2012 Based on Systematic Review and Global Reporting
Source: PLoS One. 2015 Dec 8;10(12):e0143304. doi: 10.1371/journal.pone.0143304 (PMC4672879; doi:10.1371/journal.pone.0143304)
Supplement: S5 Text — (PDF) [file pone.0143304.s010.pdf]

Newman, Lori M

From: permissions@who.int
Sent: Wednesday, October 07, 2015 12:19 PM
To: len4@cdc.gov
Cc: permissions@who.int
Subject: ID: 184371 Permission authorization for WHO copyrighted material

Follow Up Flag: Follow up
Flag Status: Flagged

Dear Dr Newman

Thank you for your request for permission to reprint and reproduce certain WHO copyrighted material .

On behalf of the World Health Organization, we are pleased to authorize your request to reproduce the WHO materials as detailed in the form below, subject to the terms and conditions of the non-exclusive licence below.

If you have questions regarding this authorization, please contact permissions@who.int.

We thank you for your interest in WHO published materials.

Kind regards,
WHO Permissions team

WORLD HEALTH ORGANIZATION (WHO)

Non-exclusive licence to use selected WHO published materials

You submitted a request, through WHO's online platform, for permission to reprint and reproduce certain WHO copyrighted material (the "Licensed Materials"). This is a legal agreement (the "Agreement") between you and WHO, granting you a licence to use the Licensed Materials subject to the terms and conditions herein.

Read this Agreement in its entirety before using the Licensed Materials.

By using the Licensed Materials, you enter into, and agree to be bound by, this Agreement.

This licence is granted only for original materials belonging to WHO. If any part of the WHO published materials you wish to reproduce are credited by WHO to a source other than WHO, those materials are not covered by this Agreement and are not part of the Licensed Materials. You are responsible for determining if this is the case, and if so, you are responsible for obtaining any necessary permission from the source of those third-party materials prior to their use.

If you enter into this Agreement on behalf of an organization, by using the Licensed Materials you confirm (represent and warrant) that you are authorized by your organization to enter into this Agreement on the organization's behalf. In such a case, the terms "you" and "your" in this Agreement refer to, and this Agreement applies to, the organization.

WHO grants this license to you based on the representations and warranties you made in the license request you submitted through WHO's online platform. If any of those representations and/or warranties are or become false or inaccurate, this license agreement shall automatically terminate with immediate effect, without prejudice to any other remedies which WHO may have.

If you have questions regarding this Agreement, please contact permissions@who.int.

1. Licence. Subject to the terms and Conditions of this Agreement, WHO grants to you a worldwide, royalty free, non-transferable, non-sublicensable, non-exclusive licence to use, reproduce, publish, and display the Licensed Materials in the manner and using the media indicated in the Permissions Request Form you submitted to WHO (the "Licensed Use"). This licence is limited to the current edition of your publication. Future editions or a different use of the Licensed Materials will require additional permission from WHO. If your request includes translation into different languages, then non-exclusive permission is hereby granted to translate the Licensed Materials into the languages indicated.

2. Retained Rights. Copyright in the Licensed Materials remains vested in WHO, and WHO retains all rights not specifically granted under this Agreement.

3. Mandatory Acknowledgement. In every instance of the Licensed Use, you must make suitable acknowledgement of WHO, either as a footnote or in a reference list at the end of your publication, as follows:

> "Reprinted from Publication title, Vol /edition number, Author(s), Title of article / title of chapter, Pages No., Copyright (Year)."

In addition, If the Licensed Materials originate from the WHO web site, you must also include the URL reference and the date accessed.

Translations of the Licensed Materials should be attributed as follows:

> "Translated with permission of the publisher from Publication title, Vol /edition number, Author(s), Title of article / title of chapter, Pages No., Year."

4. Altering or Modifying the Licensed Materials. As part of the Licensed Use, you may minimally alter or adapt figures and tables in the Licensed Materials to match the style of your publication. Any other alteration or modification of the Licensed Materials (including abbreviations, additions, or deletions) may be made only with the prior written authorization of WHO.

5. Appropriate and Prohibited Uses. You must use the Licensed Materials in a factual and appropriate context. You may not use the Licensed Materials in association with any product marketing, promotional, or commercial activities, including, without limitation, in advertisements, product brochures, company-sponsored web sites, annual reports, or other non-educational publications or distributions.

6. No WHO endorsement. You shall not state or imply that WHO endorses or is affiliated with your publication or the Licensed Use, or that WHO endorses any entity, organization, company, or product.

7. No use of the WHO logo. In no case shall you use the WHO name or emblem, or any abbreviation thereof. Notwithstanding the foregoing, if the WHO name and/or emblem appear as an integral part of the Licensed Materials (e.g. on a map) you may use the name and/or emblem in your use of the License Materials, provided the name and/or logo is not used separately from the Licensed Materials.

8. No Warranties by WHO. All reasonable precautions have been taken by WHO to verify the information contained in the Licensed Materials. However, WHO provides the Licensed Materials to you without warranty of any kind, either expressed or implied, and you are entirely responsible for your use of the Licensed Materials. In no event shall WHO be liable for damages arising from your use of the Licensed Materials.

9. Your Indemnification of WHO. You agree to indemnify WHO for, and hold WHO harmless against, any claim for damages, losses, and/or any costs, including attorneys' fees, arising in any manner whatsoever from your use of the Licensed Materials or for your breach of any of the terms of this Agreement.

10. Termination. The licence and the rights granted under this Agreement shall terminate automatically upon any breach by you of the terms of this Agreement. Further, WHO may terminate this licence at any time with immediate effect for any reason by written notice to you.

11. Entire Agreement, Amendment. This Agreement is the entire agreement between you and WHO with respect to its subject matter. WHO is not bound by any additional terms that may appear in any communication from you. This Agreement may only be amended by mutual written agreement of you and WHO.

12. Headings. Paragraph headings in this Agreement are for reference only.

13. Dispute resolution. Any dispute relating to the interpretation or application of this Agreement shall, unless amicably settled, be subject to conciliation. In the event of failure of the latter, the dispute shall be settled by arbitration. The arbitration shall be conducted in accordance with the modalities to be agreed upon by the parties or, in the absence of agreement, with the rules of arbitration of the International Chamber of Commerce. The parties shall accept the arbitral award as final.

14. Privileges and immunities. Nothing in or relating to this Agreement shall be deemed a waiver of any of the privileges and immunities enjoyed by WHO under national or international law and/or as submitting WHO to any national court jurisdiction.

***

DataCol Web: Form to request permission to reproduce or reprint WHO copyrighted material
==

ID: 184371

Section: Contact details
----

* Title
* Dr

----

* First name
* Lori
----
* Family name
* Newman
----
* Organization/affiliation
* US Centers for Disease Control and Prevention
----
* Web site address
*
----
* Type of organization
* Government
----
* If other, please specify
*
----
* Position
* Strategic Information Advisor
----
* Telephone
* +855 12 834 089
----
* Address
* United States Embassy, #1, Street 96, Sangkat Wat Phnom, Khan Daun Penh, Phnom Penh, Cambodia
----
* Country
* Cambodia
----
* Email
* len4@cdc.gov

Section: Information about WHO material to be reproduced
----
* Full title of WHO material from which the reproduction is to be made
* Classification of countries for WHO 2012 estimates of chlamydia, gonorrhoea, trichomoniasis, and syphilis.
----
* Website URL where WHO material is published
* Not currently available on web
----
* ISBN / WHO Reference Number
* No ISBN number
----
* Please select the item(s) to be reproduced
* Figure
----
* Type of reuse
* Scientific journal/book
----
* No of item(s) to be reproduced

* 5 items or less
----
* For each item, please provide a reference and page number. If entire document, please state "Entire document".
* Entire figure (map)

Section: Information about your publication
----
* Please provide the title of your publication that the above materials are to be published in
* Global Estimates of the Prevalence and Incidence of Four Curable Sexually Transmitted Infections in 2012 Based on Systematic Review and Global Reporting
----
* Publishing format
* PDF
----
* Will you be translating?
* No
----
* If yes, please indicate languages
*
----
* If web please provide URL / If other, please specify
*
----
* Number of copies (if applicable)
*
----
* Target audience and planned distribution
* PLOS One - accepted with minor revisions
----
* Planned publication/distribution date
* 4th quarter 2015
----
* If your publication or the material is to be sold, indicate the planned selling price or subscription fee
* Not for sale, open access journal
----
* Is your publication sponsored or funded by an organisation other than your own?
* No
----
* If yes, please provide additional information
*
----
* Will there be any advertising associated with your publication?
* No
----
* If yes, please provide additional information
*
----
* Subject(s) of interest that most correspond to your request
* Sexually transmitted infections
----

* Additional information about your request
*

----
* Approval
* Auto permission
----
* Latest approval modification
----
* WHO Department
* ACP, ACT

Section: Terms and conditions
----
* By submitting this request you confirm that you will abide by the terms and conditions if WHO grants you permission.
* I have read and agree with the terms and conditions

Click the following link to access a format view of this record:
http://redirect.state.sbu/?url=http://apps.who.int/datacol/survey.asp?survey_id=258&respondent_id=184371

This email was automatically sent to you by the WHO Intranet Data Collector.
The DataCol can send emails to accounts specified by the Form focalpoint.
